# Geochemical factors associated with seasonal shifts in the abalone gut microbiota across urchin-barren habitats

**DOI:** 10.3389/fmicb.2026.1817344

**Published:** 2026-05-08

**Authors:** Jae-Won Jo, Joon-Young Park, Jin-Jae Lee, Min-Jung Lee, Bong-Soo Kim

**Affiliations:** 1 Department of Life Science, Multidisciplinary Genome Institute, Hallym University, Chuncheon, Gangwon-do, Republic of Korea; 2 Department of Nutritional Science and Food Management, Ewha Womans University, Seoul, Republic of Korea; 3 Global Food and Nutrition Research Institute, Ewha Womans University, Seoul, Republic of Korea

**Keywords:** benthic herbivore, environmental changes, gut microbiota, macroalgal limitation, marine gastropod

## Abstract

The collapse of kelp forests into urchin barrens is associated with changes in seawater chemistry and macroalgal food availability, but how these environmental changes affect herbivore-associated microbiota remains poorly understood. Here, we analyzed the gut microbiota of abalone (*Haliotis discus hannai*) collected from mild and severe barren regions along the Korean coast during winter and summer, comparing wild abalones with those from aquaculture. Using 16S rRNA amplicon sequencing together with stepwise negative controls and prevalence-based decontamination, we characterized microbiota variation in relation to sampling site, season, and geochemical factors. Gut microbiota varied significantly with sampling site and multiple geochemical parameters, and differences between wild and farmed abalones were most strongly associated with suspended particulate matter. Seasonal turnover was observed in both groups, and analyses in wild abalones indicated that seasonal microbiota shifts were more closely associated with temperature-related geochemical variation than with temperature alone. Seasonal changes included a positive association between *Vibrio* abundance and chemical oxygen demand and a negative association between *Propionigenium* abundance and dissolved oxygen. The effects of barren severity were most pronounced in summer. After accounting for site differences, severe barren regions showed distinct microbiota composition, altered microbial interactions, decreased Pseudomonadota and Verrucomicrobiota, and increased Bacteroidota. PICRUSt2-based functional prediction suggested that severity-associated differences were mainly related to carbohydrate and energy metabolism, with lower predicted carbon fixation and pyruvate metabolism and higher predicted staurosporine biosynthesis in severe barren regions. These findings indicate that seasonal geochemical dynamics and barren-associated habitat degradation are associated with variation in the abalone gut microbiota, with the strongest ecological divergence occurring in summer.

## 1 Introduction

Kelp forests (macroalgal forests) serve vital roles in coastal ecosystems by contributing to fisheries production, carbon sequestration, and nutrient cycling (Krumhansl et al., 2016; Jayathilake and Costello, 2021; Eger et al., 2023). Beyond their economic value, kelp forests act as ecosystem engineers by enhancing habitat complexity, increasing species richness, and supporting marine biodiversity (Araujo et al., 2013). Despite their importance, kelp forests have declined globally, with many regions undergoing regime shifts to alternative states such as sea urchin barrens, which are characterized by the loss of macroalgae and the dominance of grazing-resistant substrates. These shifts are driven by various factors, including ocean warming, coastal pollution, and heightened grazing pressure from kelp herbivores, resulting in significant ecological and socioeconomic consequences (Coleman et al., 2008; Verges et al., 2014; Smale, 2020).

In South Korea, the expansion of barren regions has become a major ecological issue. Nationwide surveys indicate that 12,728.5 hectares (ha), or 33.6% of the total surveyed area of 37,921.4 ha, are affected by barren conditions, with the East Sea being the most severely affected region (Park and Lee, 2023). Among the key grazers in these ecosystems, abalone and sea urchin are ecologically important and represent major marine resources in South Korea (Park and Kim, 2013; Jeon et al., 2015). Abalones inhabit shallow subtidal zones and are herbivorous invertebrates that primarily feed on macroalgae (Pan et al., 2018; Kang et al., 2021). Through their grazing and interactions with other herbivores, abalone can influence kelp recruitment and the stability of kelp forest habitats (Wing et al., 2015). Although sea urchins are often considered the main drivers of kelp forest decline and barren formation, increasing evidence suggests that herbivorous gastropods, including abalone, may also play a role in maintaining or reinforcing barren states under certain ecological conditions (Wing et al., 2015; Yatsuya and Matsumoto, 2023). In fact, the excessive release of abalone without considering the carrying capacity of rocky reef habitats may worsen kelp forest degradation in South Korea (Kang, 2010). One possible explanation is that adult abalones feed mainly on drift macroalgae but may occasionally graze attached algae when drift resources are limited; thus, stock release could potentially increase local herbivory pressure on remnant macroalgal beds in degraded habitats, although direct evidence that abalone release accelerates kelp forest degradation remain limited (Jenkins, 2004; Zeeman et al., 2012). These findings highlight the need for improved understanding of the physiological and ecological responses of herbivorous gastropods, particularly abalones, across varying degrees of barren formation and persistence.

The gut microbiome is crucial for host physiology, contributing to nutrient utilization, immune regulation, and metabolic homeostasis (Oliphant and Allen-Vercoe, 2019; Zheng et al., 2020). In marine organisms, gut microbiota is highly dynamic and responds sensitively to environmental changes and dietary shifts (Pierce and Ward, 2019; Wang X. Z. et al., 2020; Hou et al., 2024). In abalones, seawater temperature, nutrient composition, salinity, pH, and diet have been reported to shape gut microbiota composition (Wang X. Z. et al., 2020; Li et al., 2025a; Ma et al., 2025). Abalone gut microbiota is typically dominated by a relatively simple set of anaerobic or facultative anaerobic microbes, including *Psychrilyobacter*, *Mycoplasma*, and *Vibrio*, which have been repeatedly reported across multiple abalone systems (Gobet et al., 2018; Danckert et al., 2021; Zhang et al., 2022). Moreover, a previous study in *Haliotis discus hannai* showed that gut microbiota shifts markedly during ontogenetic and dietary transition, particularly toward algal polysaccharide-degrading bacteria, whereas other studies have reported additional restructuring associated with temperature, pH, dissolved oxygen, and feed type, supporting the hypothesis that ecological gradients can reshape abalone microbiota with potential consequences for host nutrition and health (Wang X. Z. et al., 2020; Zhang et al., 2022; Guo et al., 2024). Aquaculture introduces distinct diets and environmental microbial exposures compared to natural habitats, which can systematically alter gut microbiota and its seasonal dynamics (Zhang et al., 2022; Sun et al., 2024; Sawant et al., 2025). Therefore, life type (wild vs. farmed) should be considered an important explanatory factor in gut microbiota studies. Our recent study further showed that sea urchin gut microbiota shift according to barren severity, emphasizing the sensitivity of host-associated microbial communities to ecosystem-level disturbances (Park et al., 2023). However, despite the ecological and economic importance of abalone in kelp-barren systems, the relationship between abalone gut microbiota and barren severity remains largely unexplored, particularly across seasonal and regional geochemical gradients that coexist with barren formation and persistence. In this study, barren severity was operationally defined based on the site-level cover of crustose coralline algae, a characteristic substrate of barren habitats, enabling sites to be classified along a degradation gradient (Korea Fisheries Resources Agency [FIRA], 2016; Park et al., 2023).

To address this gap, we investigated how seasonal and regional geochemical gradients, barren severity, and life type (wild vs. farmed) are associated with variation in the gut microbiota of *Haliotis discus hannai* across urchin-barren habitats. Our objectives were to characterize taxonomic variation in relation to geochemical conditions, season, barren severity, and life type; to identify severity-associated taxa and microbial interaction patterns; and to assess whether predicted microbial functions also differed across ecological contexts. We hypothesized that geochemical and dietary constraints would be associated with directional shifts in gut microbiota, with stronger divergence under severe barren conditions and during summer, when macroalgal limitation and host feeding demand are expected to be greatest. We further expected that these shifts would be accompanied by differences in predicted metabolic potential, particularly in functions related to nutrient and energy metabolism. By clarifying these patterns, this study aims to provide an ecological framework for understanding how habitat degradation shapes host-associated microbiota and to generate baseline knowledge relevant to future ecological monitoring and abalone aquaculture management under environmentally degraded conditions.

## 2 Materials and methods

### 2.1 Sample and geochemical data collection

**Figure 1:**
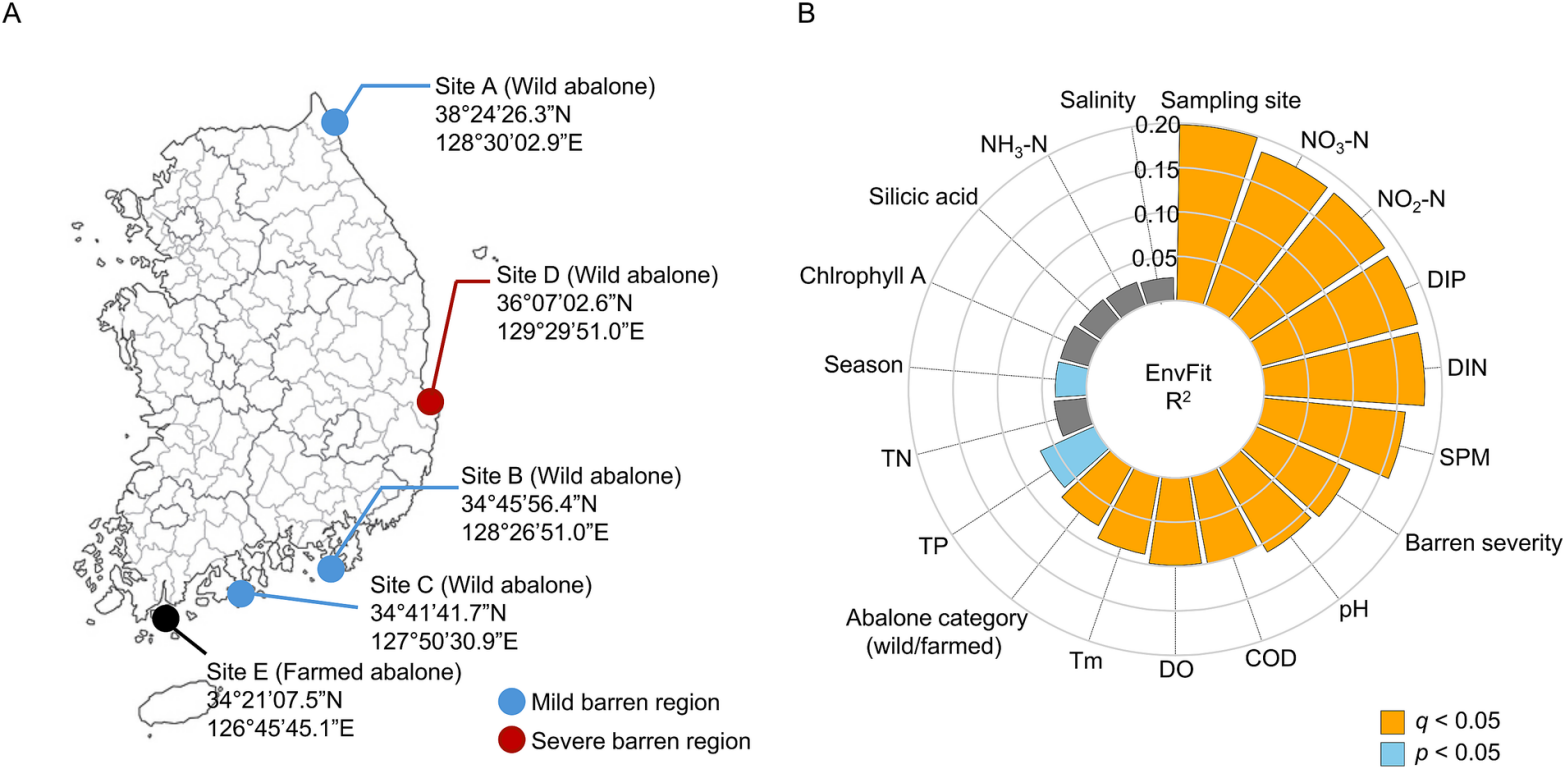
Sampling design and environmental factors associated with variation in the abalone gut microbiota. **(A)** Wild abalones were randomly collected (5–6 individuals per site) in February and August from three mild barren regions (Sites A–C; blue circles) and one severe barren region (Site D; red circle). Farmed abalones were collected from an aquaculture site (Site E; black circle) in both February and August (*n* = 24 for each season). **(B)** Environmental and geochemical factors associated with variations in the abalone gut microbiota, as identified by EnvFit. The circular bar plot displays *R*^2^ values for each variable; variables with *q* < 0.05 are highlighted in orange, and those with *p* < 0.05 are highlighted in blue.

Wild abalone (*H. discus hannai*, *n* = 47) were collected in February and August 2022 from three regions classified as mild barren (Goseong, Tongyeong and Yeosu; *n* = 35) and one region classified as severe barren (Pohang; *n* = 12) (Figure 1 and Supplementary Table S1). Farmed abalones (*n* = 48) were obtained from an aquaculture facility in Wando during the same period. Sampling sites were selected based on regional urchin-barren status reports, annual statistics on abalone production, and the operational status of cage-aquaculture facilities (Supplementary Tables S2–S4). Because abalone metabolic demand and coastal environmental conditions vary with seawater temperature (Kang et al., 2019; Seo and Koo, 2023), sampling was conducted in both February and August to assess seasonal differences in host-associated microbiota and geochemical conditions. Information on barren status for each site was provided by the Korea Fisheries Resources Agency (FIRA) (Supplementary Table S2). Barren severity was categorized based on the cover of crustose coralline algae in the survey region (normal: <40%; mild: 40–79%; severe: ≥80%) (Korea Fisheries Resources Agency [FIRA], 2016; Park et al., 2023). Abalones (*n* ≥ 5) were randomly collected at each site and transported to the laboratory on ice in sterile containers. Upon arrival at the laboratory, whole gut samples (including gut tissue and contents) were dissected as quickly as possible using sterilized knives, forceps, and scissors, and transferred into sterile 2-ml tubes. Whole gut samples were not preserved in ethanol; instead, they were stored at −80 °C until DNA extraction. DNA extraction was performed as promptly as possible and within 2 weeks of whole gut dissection. When immediate downstream processing was not possible, both dissected gut samples and extracted DNA were stored at −80 °C until the next step. Geochemical data corresponding to the sampling periods and regions were obtained from the Marine Environmental Information System operated by the Korea Ministry of Oceans and Fisheries [MOF] (2022) (Supplementary Table S1).

### 2.2 DNA extraction and 16S rRNA amplicon sequencing

Metagenomic DNA was extracted from abalone gut samples using the RNeasy PowerMicrobiome Kit (Qiagen, Inc., Valencia, CA, United States). Extracted DNA was further purified using the DNeasy PowerClean Pro Cleanup Kit (Qiagen) following the manufacturer’s instructions. The V1–V3 regions of the 16S rRNA gene were amplified using the following primers: forward, 5′-TCG TCG GCA GCG TCA GAT GTG TAT AAG AGA CAG AGA GTT TGA TCM TGG CTC AG-3′; reverse, 5′-GTC TCG TGG GCT CGG AGA TGT GTA TAA GAG ACA GAT TAC CGC GGC TGC TGG-3′. PCR amplification was performed according to the protocol for preparing a 16S metagenomic sequencing library, as previously described (Lee et al., 2022; Park et al., 2023). Briefly, the first round of PCR was carried out in a final volume of 25 μL containing 0.2 μM of each primer, 1.25 U of Ex Taq polymerase (Takara Bio, Shiga, Japan), 2.5 μL of 10 × Ex Taq buffer, 4 μL of dNTP mixture, and 2.5 μL of template DNA (≥ 20 ng/μL), using a C1000 thermal cycler (Bio-Rad, Hercules, Germany). The amplification conditions were as follows: initial denaturation at 95 °C for 3 min; 25 cycles of 95 °C for 30 s, 55 °C for 30 s, and 72 °C for 30 s; and a final extension at 72 °C for 5 min. Amplicons were purified and size-selected using HiAccuBead (AccuGene, Inc., San Diego, CA, United States). Index PCR was then performed using the Nextera XT v2 Index Kit (Illumina, Inc., San Diego, CA, United States) under the following conditions: initial denaturation at 95 °C for 3 min, followed by 8 cycles of 95 °C for 30 s, 55 °C for 30 s, and 72 °C for 30 s, with a final extension at 72 °C for 5 min. The indexed PCR products were purified and size-selected again using HiAccuBead. Amplicon libraries were quantified using the Qubit™ dsDNA HS Assay Kit (Thermo Fisher Scientific, Inc., Waltham, MA, United States), pooled at equimolar concentrations, and sequenced on an Illumina MiSeq platform (300-bp paired-end reads) following the manufacturer’s guidelines.

Due to the susceptibility of sequencing-based studies of low-biomass samples to contamination, which can bias results (Glassing et al., 2016; Eisenhofer et al., 2019), seven negative controls were included throughout the experimental process and sequenced alongside the abalone samples. These negative controls comprised an empty sampling container, an empty sample storage tube, a stainless-steel tray used for gut dissection, and DNA-free water controls introduced during DNA extraction, purification, and library preparation.

### 2.3 Quantification of bacterial loads by quantitative real-time PCR

Bacterial loads in each sample were quantified in triplicate using quantitative real-time PCR targeting the 16S rRNA gene, as previously described (Lee et al., 2022; Park et al., 2023). DNA extracted from each sample was amplified in a 25-μL reaction mixture containing 12.5 μL of 2 × TB Green Premix Ex Taq (Tli RNaseH Plus, TaKaRa Bio), 20 μM of each primer (340F: 5′-TCC TAC GGG AGG CAG CAG-3′; 518R: 5′-ATT ACC GCG GCT GCT GG-3′), and 1 μL of template DNA (10-fold serial dilutions of sample DNA) or distilled water (no-template control). Amplification was conducted using a Thermal Cycle Dice Real-Time System III (Takara Bio) under the following conditions: initial denaturation at 95 °C for 30 s, followed by 40 cycles of denaturation at 95 °C for 5 s and annealing at 60 °C for 30 s. Standard curves were generated from 10-fold serial dilutions (1 × 10^−1^ to 1 × 10^−6^) of 16S rRNA gene copy numbers derived from *Escherichia coli* K12 w3110. Bacterial loads were estimated by comparing threshold cycle (Ct) values to the standard curves generated in parallel, with regression coefficients (*r*^2^) for all standard curves being ≥0.99.

### 2.4 Amplicon sequencing data processing

Raw sequence reads were processed using the QIIME2 pipeline (Bolyen et al., 2019). Reads were imported as QIIME2 artifacts using the *“qiime tools import”* function, and demultiplexing summaries were generated using *“qiime demux summarize.”* Quality filtering, denoising, paired-end merging, and chimera removal were performed using DADA2 implemented in QIIME2 (Callahan et al., 2016). Quality control outputs were visualized using *“qiime metadata tabulate.”* Amplicon sequence variants (ASVs) were taxonomically assigned using the classify-sklearn method against the EzBioCloud database (Yoon et al., 2017).

### 2.5 Microbiota analyses

#### 2.5.1 Decontamination and feature filtering

To eliminate potential contaminants, we utilized the R package “decontam” based on sequences obtained from negative controls. Contaminant identification was performed using the *“isContaminant”* function (prevalence method, threshold = 0.2). Following decontamination, features were retained for downstream analyses if they met both prevalence (>20% of samples) and relative abundance (>0.001%) criteria.

#### 2.5.2 Ordination and environmental association analysis

Microbiota dissimilarity was calculated using Bray–Curtis distance, and β-diversity was visualized through non-metric multidimensional scaling (NMDS). Environmental and sampling variables, including seawater temperature, salinity, pH, dissolved oxygen, chemical oxygen demand, ammonium-nitrogen, nitrite-nitrogen, nitrate-nitrogen, dissolved inorganic nitrogen, dissolved inorganic phosphorus, total nitrogen, total phosphorus, silicic acid, chlorophyll A, suspended particulate matter, life type, season, sampling site, and barren severity, that influence gut microbiota were assessed using the *“envfit”* function in the R package vegan (v.2.5–7). Permutational multivariate analysis of variance (PERMANOVA) was conducted to determine significance, with variables having *p* < 0.05 considered significant.

#### 2.5.3 Functional prediction and KO-level analysis

Functional profiles of microbiota were predicted using Phylogenetic Investigation of Communities by Reconstruction of Unobserved States (PICRUSt2) (Douglas et al., 2020). KEGG Orthology (KO) abundances were inferred from ASV sequences generated in QIIME2, and these functional profiles were normalized using cumulative sum scaling. Differences in KO gene families were visualized using volcano plots, where the *x*- and *y*-axes represent log_2_ fold-change and -log_10_(*p-*value), respectively, with *p-*values computed using MaAsLin2. KO gene families with |log_2_fold-change| > 0.5 and *p* < 0.01 were considered significant.

#### 2.5.4 Generalized linear models

Generalized linear models (GLMs) were fitted using the *“glm”* function in the R package stats (v.4.1.1) to test for (i) differences in Shannon diversity and bacterial load between groups and (ii) differences in geochemical factors between groups. The Shapiro–Wilk test guided the choice of model family: Gaussian models were used when *p* > 0.05, whereas Gamma models with a log link were used when *p* < 0.05. Potential confounders, such as sampling site and/or season, were included as covariates when appropriate.

#### 2.5.5 MaAsLin2 for different abundances and associations

Differential abundance and associations between geochemical factors and microbial genera were evaluated using Multivariate Association with Linear Models 2 (MaAsLin2) (Mallick et al., 2021). Features were included if present at ≥ 0.1% relative abundance in ≥10% of samples (min_abundance = 0.1%, min_prevalence = 0.1). All other parameters were set to default. Results with *p* < 0.05 were considered significant.

#### 2.5.6 Mediation analysis

Mediation analysis was performed using the *“mediate”* function from the R package mediation (Tingley et al., 2014) to investigate whether geochemical factors mediated the effect of seawater temperature or season on variations in gut microbiota. The total effect was divided into direct and indirect effects. When season was considered as exposure, sampling site was included as a covariate. Statistical significance was evaluated using non-parametric bootstrapping via 1,000 simulations (boot = TRUE, sims = 1,000).

#### 2.5.7 Random forest classification

Random forest analysis was conducted using the R package randomForest to identify taxonomic and functional features that differentiate mild from severe barren regions. The data were divided into training (70%) and testing (30%) sets. Models were trained with 1,000 trees (ntree = 1,000), and the *mtry* parameter was set to its default value, which is square root of the number of features. Candidate features were ranked through 10-fold cross-validation using the *“rfcv”* function. Feature importance was assessed by calculating the mean decrease in Gini with the *“importance”* function. Of the candidate discriminatory features, only those exhibiting statistically significant group differences were retrained in a refined model. Model performance was evaluated on the testing set using a receiver operating characteristic (ROC) curve, and the area under the curve (AUC) was computed with the *“roc”* function from the R package pROC. A 95% confidence interval (CI) for the AUC was estimated using the *“ci.auc”* function with 2,000 bootstrap replicates.

#### 2.5.8 Co-occurrence network analysis

Gut microbial co-occurrence networks were constructed separately for mild and severe barren regions within the August dataset. Taxa with a relative abundance of ≥ 0.01% and a prevalence of ≥ 30% were retained. Pairwise correlations were estimated using FastSpar, applying Pearson correlation and 1,000 bootstraps (Watts et al., 2019). Pseudo *p values* were calculated as the proportion of bootstrapped datasets that yielded correlations at least as extreme as the observed values. Significant edges were defined as having *p* < 0.05 and |correlation| > 0.6. The resulting adjacent structure was used to visualize the networks. Keystone taxa were identified as the top five nodes ranked by PageRank centrality. Network modularity and module counts were determined using the fast-greedy clustering algorithm from the *“cluster_fast_greedy”* function in the R package igraph.

### 2.6 Statistical analysis

Differences in microbial features between the two groups were tested using the Wilcoxon rank-sum test in R. β-diversity patterns were visualized using NMDS plots based on Bray–Curtis dissimilarity, and significance was determined using PERMANOVA with 1,000 permutations via the *“adonis”* function in the R package vegan (v.2.5–7). When necessary, covariates such as season and/or sampling site were included in the PERMANOVA analysis to account for potential confounding effects. For analyses that did not involve covariate adjustment, Spearman’s rank correlation was used to identify relationships between microbial features and geochemical variables. Multiple-testing correction was performed using the Benjamini–Hochberg false discovery rate (FDR) method, and adjusted *p*-values are reported as *q*-values.

## 3 Results

### 3.1 Geochemical correlates of abalone gut microbiota across sites, seasons, and life types

Wild abalones (*n* ≥ 5 per site) were collected from three mild barren sites and one severe barren site in South Korea, whereas farmed abalones (*n* ≥ 24) were sampled from a Wando aquaculture facility in February and August 2022 (Figure 1A and Supplementary Table S1). To reduce potential bias from contaminants commonly associated with sequence-based studies of low-biomass samples (Glassing et al., 2016; Eisenhofer et al., 2019), negative controls were processed concurrently. This resulted in the identification and removal of 98 features considered putative contaminants, yielding 841 retained features (Supplementary Figure S1). Following quality filtering, gut microbiota from 95 abalones were included in subsequent analyses.

The effects of geochemical parameters and sampling metadata on gut microbiota variation were determined using EnvFit with FDR correction (Figure 1B). Gut microbiota showed significant associations with the sampling site (*R*^2^ = 0.198, *q* = 0.001) and nutrient-related variables such as nitrate-nitrogen (NO_3_-N; *R*^2^ = 0.185, *q* = 0.001), nitrite-nitrogen (NO_2_-N; *R*^2^ = 0.184, *q* = 0.001), dissolved inorganic phosphorus (DIP, *R*^2^ = 0.182, *q* = 0.001), and dissolved inorganic nitrogen (DIN; *R*^2^ = 0.180, *q* = 0.001). Suspended particulate matter (SPM) also exhibited a strong association (*R*^2^ = 0.160, *q* = 0.001), followed by barren severity (mild vs. severe; *R*^2^ = 0.116, *q* = 0.002) and physicochemical variables including pH (*R*^2^ = 0.111, *q* = 0.010), chemical oxygen demand (COD; *R*^2^ = 0.100, *q* = 0.016), dissolved oxygen (DO; *R*^2^ = 0.099, *q* = 0.015), and seawater temperature (Tm; *R*^2^ = 0.089, *q* = 0.019). Abalone category (wild vs. farmed; *R*^2^ = 0.076, *q* = 0.003) was also significantly associated with microbiota variation, although they explained a smaller proportion of the total variance. Sampling season (*R*^2^ = 0.035, *p* = 0.042, *q* = 0.061) and total phosphorus (TP; *R*^2^ = 0.067, *p* = 0.042 *q* = 0.061) showed nominal associations and were not significant after FDR correction. These findings indicate that site-level conditions, as well as nutrient- and particle-related geochemical factors, are major correlates of abalone gut microbiota.

### 3.2 Aquaculture-associated SPM correlates with gut microbiota divergence between wild and farmed abalones

**Figure 2:**
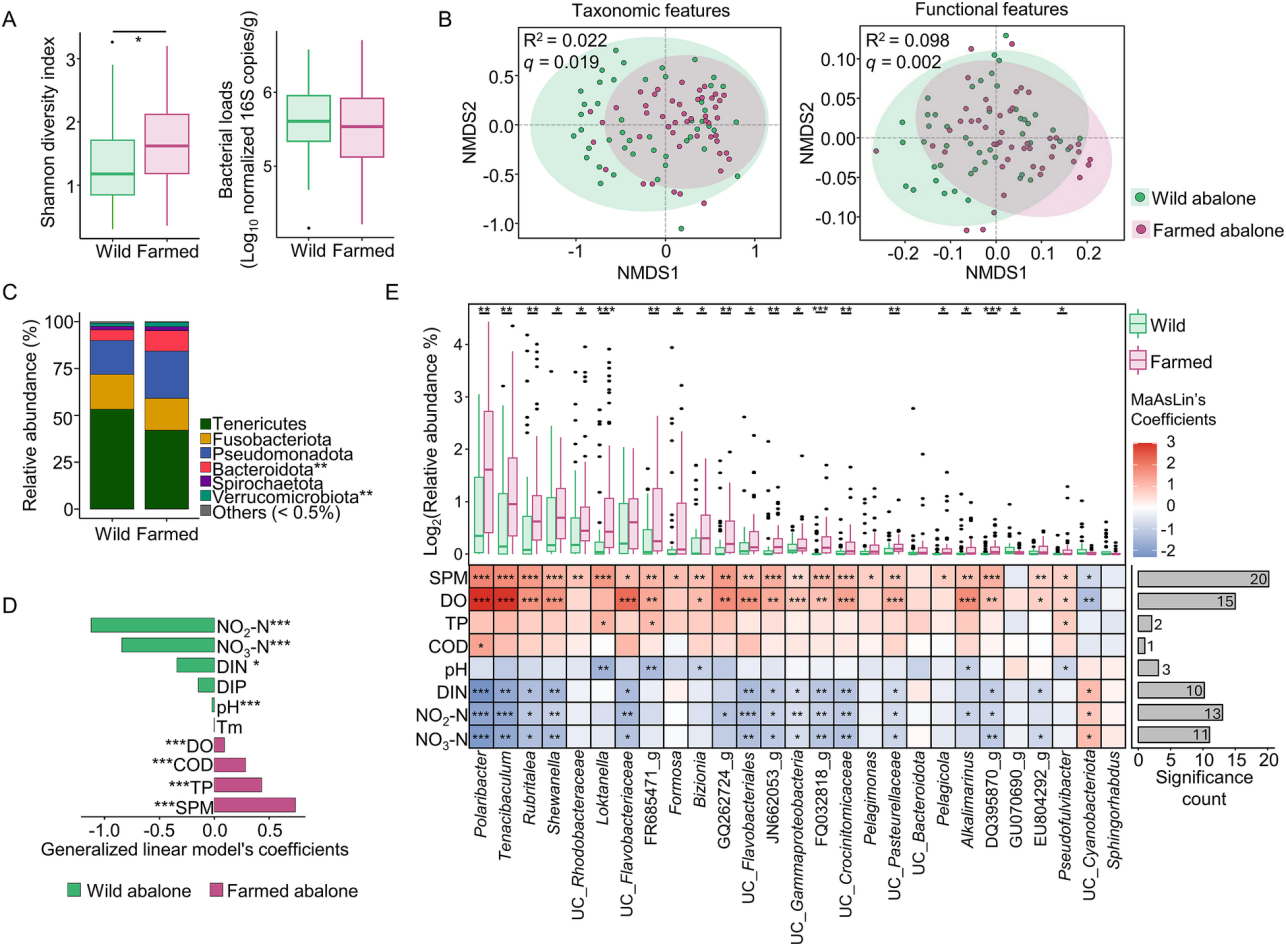
Differences in gut microbiota between wild and farmed abalone. **(A)** Shannon diversity and bacterial load in wild (*n* = 47) and farmed (*n* = 48) abalone, tested using GLM with season as a covariate. **(B)** Taxonomic and functional features of gut microbiota compared between wild and farmed abalone using an NMDS plot based on Bray–Curtis dissimilarity. Group differences were evaluated using PERMANOVA. **(C)** Phylum-level composition of gut microbiota in wild and farmed abalones, with phyla having < 0.5% relative abundance in both groups combined into “Others.” Differential abundance was analyzed using MaAsLin2, with adjusting for season. **(D)** Geochemical factors that differed between wild and farmed environments, identified by GLMs, also adjusted for season. Negative and positive coefficients indicate higher values in wild and farmed habitats, respectively. **(E)** Heatmap showing associations between 27 genera that showed nominal differences between wild and farmed abalones (MaAsLin2, *p* < 0.05), 21 of which remained significant after FDR correction (*q* < 0.05), and geochemical factors that varied between the two environments. The bar plot indicates the number of genera significantly linked to each geochemical factor. *q < 0.05, **q < 0.01, ***q < 0.001.

To investigate gut microbiota differences between wild and farmed abalones, we compared diversity and composition while controlling for season and sampling site (Figure 2). Gut microbial diversity was significantly higher in farmed than in wild abalones (*q* = 0.020), whereas total bacterial loads did not differ between the groups (Figure 2A). Both taxonomic and functional profiles differed significantly between wild and farmed abalones (Figure 2B; PERMANOVA *q* < 0.05). MaAsLin2 analysis identified phylum-level shifts, with significant differences in the relative abundance of Bacteroidota (*q* = 0.002) and Verrucomicrobiota (*q* = 0.004) after adjusting for season and sampling site (Figure 2C).

We next evaluated the geochemical correlates of the microbiota differences between wild and farmed abalones using two complementary approaches. EnvFit identified geochemical variables that aligned with ordination patterns of gut microbiota, whereas GLMs independently tested their associations (Figure 2D). In farmed abalones, both approaches consistently highlighted SPM, TP, COD, and DO as significant correlates of microbiota variation (GLM *q* < 0.001; EnvFit *q* < 0.05). By contrast, in wild abalones, significant covariables included NO_3_-N, NO_2_-N, DIN, and pH (GLM *q* < 0.05; EnvFit *q* < 0.05). At the genus level, 21 genera showed significant differences between wild and farmed abalones after FDR correction (Supplementary Table S5; MaAsLin2 *q* < 0.05), and correlation analysis indicated that SPM was the most pervasive associated factor, showing significant association with 20 genera (Figure 2E; Spearman’s *q* < 0.05). These results suggest that aquaculture-associated particulate conditions (SPM) are strongly linked to gut microbiota divergence between wild and farmed abalones.

To assess whether these patterns were influenced by season, we conducted separate analyses for winter (February) and summer (August). In winter, neither microbial diversity nor bacterial load differed significantly between wild and farmed abalones (*q* > 0.05), although microbiota composition differed significantly (Supplementary Figures S2A,B; PERMANOVA *q* = 0.003). At the phylum level, Tenericutes differed between the two groups (Supplementary Figure S2C; Wilcoxon *q* = 0.007). Consistent with the global analysis, SPM, together with TP and DO, remained significantly associated with gut microbiota variation in farmed abalones, whereas NO_3_-N, NO_2_-N, and DIN were associated with gut microbiota variation in wild abalones (Supplementary Figure S2D; GLM *q* < 0.05; EnvFit *q* < 0.05). At the genus level, five genera differed significantly between groups after FDR correction (Supplementary Table S6; *q* < 0.05), and SPM showed the strongest genus-level associations, being significantly correlated with seven genera (Supplementary Figure S2E; Spearman’s *q* < 0.05).

In summer, gut microbial diversity was higher in farmed than in wild abalones (*q* = 0.048), whereas bacterial loads and microbiota composition did not differ significantly between the groups (Supplementary Figures S3A,B; GLM *q* > 0.05 and PERMANOVA *q* > 0.05, respectively). At the phylum level, Verrucomicrobiota differed between the groups (Supplementary Figure S3C; Wilcoxon *q* = 0.014). In farmed abalones, SPM, TP, COD, and DO were significantly associated with gut microbiota variation, whereas NO_3_-N, NO_2_-N, and pH were associated with gut microbiota variation in wild abalones (Supplementary Figure S3D; GLM *q* < 0.05; EnvFit *q* < 0.05). Twenty-five genera differed significantly between groups in summer (Supplementary Table S7; Wilcoxon *q* < 0.05), and SPM again identified as the dominant associated factor, showing significant associations with 46 genera (Supplementary Figure S3E; Spearman’s *q* < 0.05). Overall, these findings suggest that divergence in gut microbiota between wild and farmed abalones is consistently linked to a common set of geochemical factors across both seasons, with SPM showing the broadest genus-level associations.

### 3.3 Seasonal shifts in gut microbiota are associated with geochemical variation linked to seawater temperature

**Figure 3:**
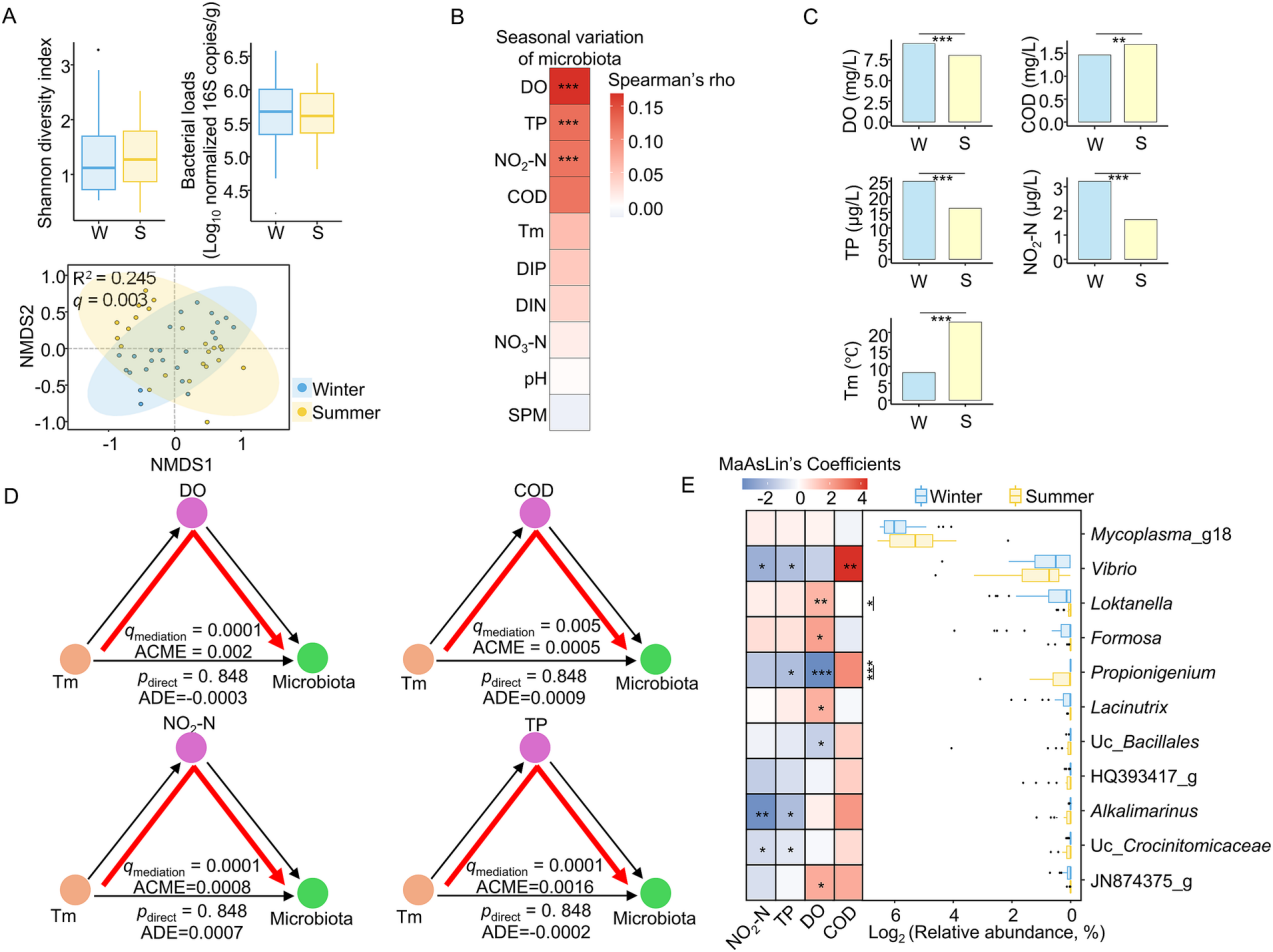
Seasonal gut microbiota turnover in wild abalones and associations with temperature-related geochemical variation. **(A)** Shannon diversity, bacterial load, and microbiota composition (Bray–Curtis dissimilarity) of wild abalone gut microbiota in winter (February) versus summer (August). Diversity and bacterial load were tested using GLM, and microbiota composition was compared using PERMANOVA with sampling site as a covariate. **(B)** Spearman correlations between Bray–Curtis dissimilarity and pairwise differences in geochemical variables, selected based on significant EnvFit results. **(C)** Seasonal variations in geochemical variables, assessed by GLMs and adjusted for sampling site. **(D)** Analysis evaluating whether DO, TP, NO₂-N, and COD statistically account for the association between seawater temperature and gut microbiota dissimilarity. Pairwise temperature differences served as the exposure, pairwise differences in each geochemical factor as covariables, and Bray–Curtis dissimilarity as the outcome. **(E)** Genera showing nominal seasonal differences in MaAsLin2 (*p* < 0.05) and their associations with geochemical factors, with sampling site included as a covariate. All genera with nominal significant seasonal differences were used for the association analysis. *q < 0.05, **q < 0.01, ***q < 0.001.

Although sampling season itself showed only a nominal association in the global EnvFit analysis (Figure 1B), several geochemical variables and Tm were significantly associated with gut microbiota variation. To minimize potential confounding from life type and aquaculture-specific effects, we focused on wild abalones to examine whether seasonal differences in microbiota were accompanied by temperature-related geochemical variation. We found no significant differences in microbial diversity or total bacterial loads between winter and summer (*q* > 0.05), although microbial composition differed significantly between season (Figure 3A; PERMANOVA *q* = 0.003).

To determine the geochemical factors associated with this seasonal variation, we selected significant variables using EnvFit and further evaluated their associations using Spearman’s correlation analysis (Figure 3B). Variables significantly associated with gut microbiota variation included DO (Spearman’s rho = 0.169, *q* < 0.001), TP (rho = 0.125, *q* < 0.001), and NO₂-N (rho = 0.121, *q* < 0.001). These variables also showed clear seasonal differences (Figure 3C; *q* < 0.01). Tm and COD were higher in summer (GLM *q* = 0.011), whereas DO, TP, and NO₂-N were lower in summer (GLM *q* < 0.001), after adjustment for sampling site.

Previous studies have suggested that seasonal temperature dynamics may influence broader geochemical changes in coastal waters (Hui et al., 2020; Li et al., 2025b). In the wild-only seasonal analysis, however, Tm showed a weaker association with microbiota variation than several geochemical factors and was not significant after FDR correction (Figure 3B). We therefore further analyzed whether seasonal microbiota shifts were more strongly associated with Tm-related geochemical covariables. This analysis showed that these covariables accounted for a substantial proportion of the observed seasonal microbiota variation (*q* < 0.01), whereas the direct association with Tm alone was not significant (Figure 3D; average direct effect *p* > 0.05).

At the taxonomic level, two genera showed significant seasonal differences after FDR correction (Supplementary Table S8; *q* < 0.05), whereas 11 genera showed nominal seasonal difference after adjustment for sampling site (MaAsLin2 *p* < 0.05). Exploratory analysis of these 11 genera revealed broadly similar associations with the geochemical variables shown in Figure 3E. The strongest positive association was observed between *Vibrio* and COD (MaAsLin2 coefficient = 4.417, *q* = 0.002), whereas the strongest negative association was found between *Propionigenium* and DO (coefficient = −3.639, *q* = 0.001). Overall, these results suggest that seasonal shifts in the gut microbiota of wild abalones are more closely associated with geochemical variation linked to seawater temperature than with temperature alone.

We next examined whether similar seasonal patterns were present in farmed abalone (Supplementary Figure S4). As in wild abalones, microbial diversity and bacterial loads in farmed abalone did not differ significantly between seasons (GLM *q* > 0.05). However, microbial composition differed significantly by season (Supplementary Figure S4A; PERMANOVA *q* = 0.005). Forty-one genera showed significant seasonal differences (Supplementary Figure S4B and Supplementary Table S9; Wilcoxon *q* < 0.05). Ten geochemical variables associated with gut microbiota variation also differed between seasons (Supplementary Figure S4C). COD, Tm, and TP were higher in summer and positively correlated with genera enriched in that season, whereas other geochemical factors were associated with genera enriched in winter (Supplementary Figure S4D). These findings indicate that farmed abalones also display seasonal shifts in gut microbiota in line with seasonal geochemical changes.

### 3.4 Differences in gut microbiota associated with barren severity are most pronounced in summer

Because sampling site explained a larger portion of gut microbiota variation than barren severity in the global model (Figure 1B), we first examined within-season patterns to differentiate variability among the mild barren sites (A–C) from the contrast between these mild sites and the severe barren region represented by Site D. In winter, neither microbial diversity nor bacterial loads showed significant differences after FDR correction, either among the mild sites (A–C) or between the mild sites (A–C) and severe site (D) (Supplementary Figure S5A). By contrast, during summer, both microbial diversity and bacterial loads exhibited a greater divergence between mild (A–C) and severe (D) regions than among the mild sites themselves (Supplementary Figure S5B; *q* < 0.05).

Microbiota showed no clear separation among mild sites in winter, and the severe site did not differ significantly from the mild sites (Supplementary Figure S5C). However, in summer, the composition of microbiota differed significantly between mild and severe regions (Supplementary Figure S5D; PERMANOVA *q* = 0.003). Pairwise comparisons revealed that the severe site (D) differed from sites B (*q* = 0.006) and C (*q* = 0.017), and that sites B and C also differed from each other (*q* = 0.004), indicating measurable heterogeneity even among mild sites. Geographic distance did not clearly explain these patterns (Figure 1A). By contrast, algal community composition correlated with microbiota dissimilarity only in summer (Supplementary Table S10 and Supplementary Figures S5E,F; Spearman’s rho = 0.238, *q* = 0.0001), suggesting a season-dependent dietary effect. These results support the need to adjust for sampling site when analyzing gut microbiota differences associated with barren severity in summer.

**Figure 4:**
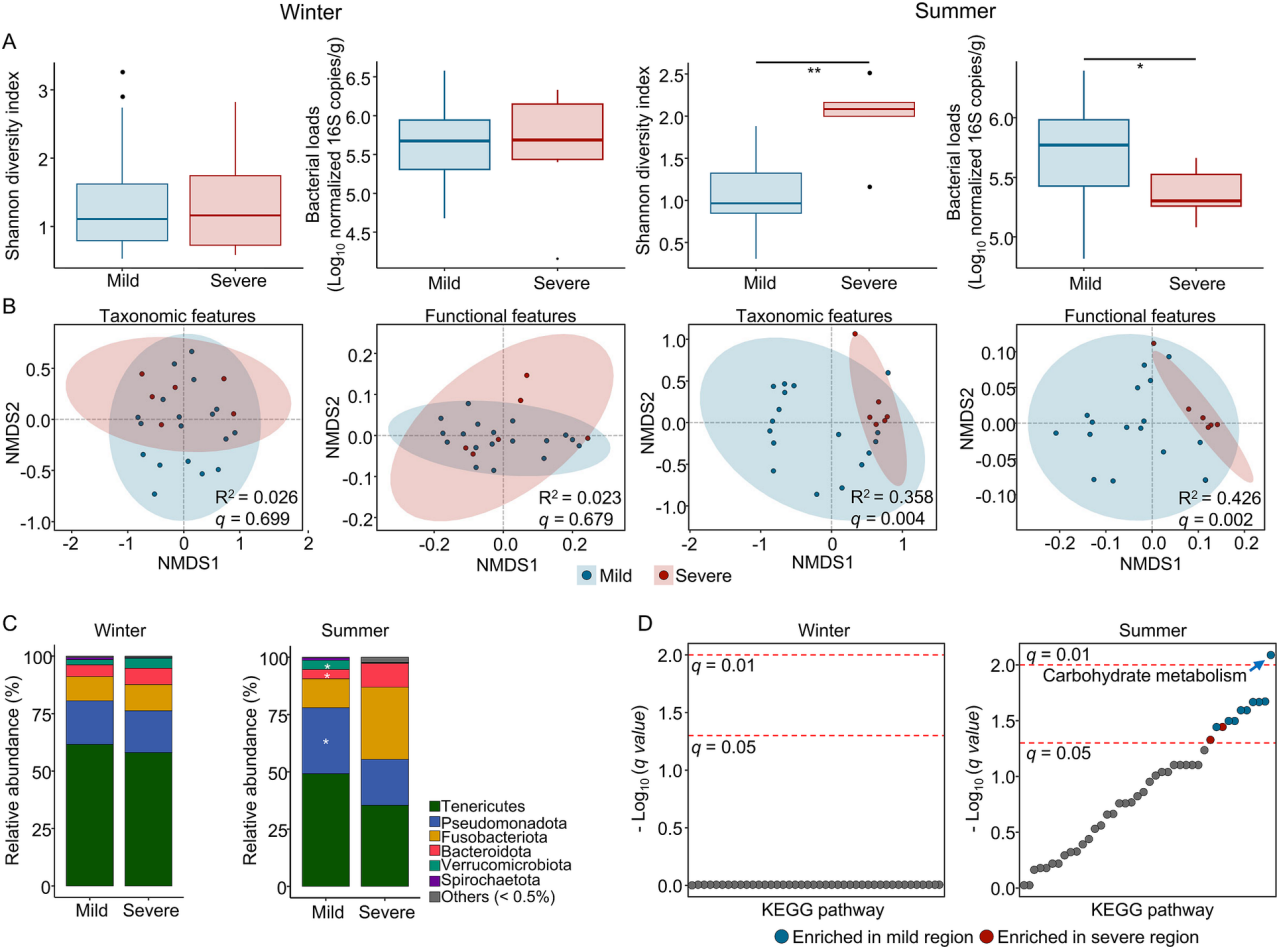
Season-dependent differences in gut microbiota by barren severity. **(A)** Shannon diversity and bacterial load (log_10_ 16S rRNA gene copies/g) in wild abalones from mild versus severe barren regions during winter and summer. Significance was assessed using GLM. **(B)** NMDS ordination of taxonomic and predicted functional features based on Bray–Curtis dissimilarity; group differences were evaluated using PERMANOVA. **(C)** Phylum-level composition in mild and severe barren regions within each season; taxa with < 0.5% relative abundance were grouped as “Others.” **(D)** Predicted KEGG pathway profiles (2nd category) compared between mild and severe barren regions for each season; carbohydrate metabolism exhibited the most significant difference in summer (*q* = 0.0081). All comparisons were adjusted for sampling site. *q < 0.05, **q < 0.01, ***q < 0.001.

After accounting for sampling site, the abalone gut microbiota still differed significantly between mild and severe barren regions in summer. Both microbial diversity (GLM *q* = 0.0004) and bacterial loads (*q* = 0.023) differed between the groups (Figure 4A), and both taxonomic and PICRUSt2-inferred functional profiles were differentiated by barren severity (Figure 4B; PERMANOVA *q* < 0.01). At the phylum level, Pseudomonadota (MaAsLin2 *q* = 0.027) and Verrucomicrobiota (*q* = 0.027) declined in the severe region, whereas Bacteroidota (*q* = 0.011) increased (Figure 4C). No significant differences associated with severity were found in winter. In summer, a greater number of predicted KEGG pathways differed by barren severity, with carbohydrate metabolism showing the most significant difference (Figure 4D; MaAsLin2 *q* = 0.0081). These results indicate that the divergence of the abalone gut microbiota associated with barren severity is most pronounced in summer.

### 3.5 Summer severity-associated taxonomic and functional signatures of the abalone gut microbiota

**Figure 5:**
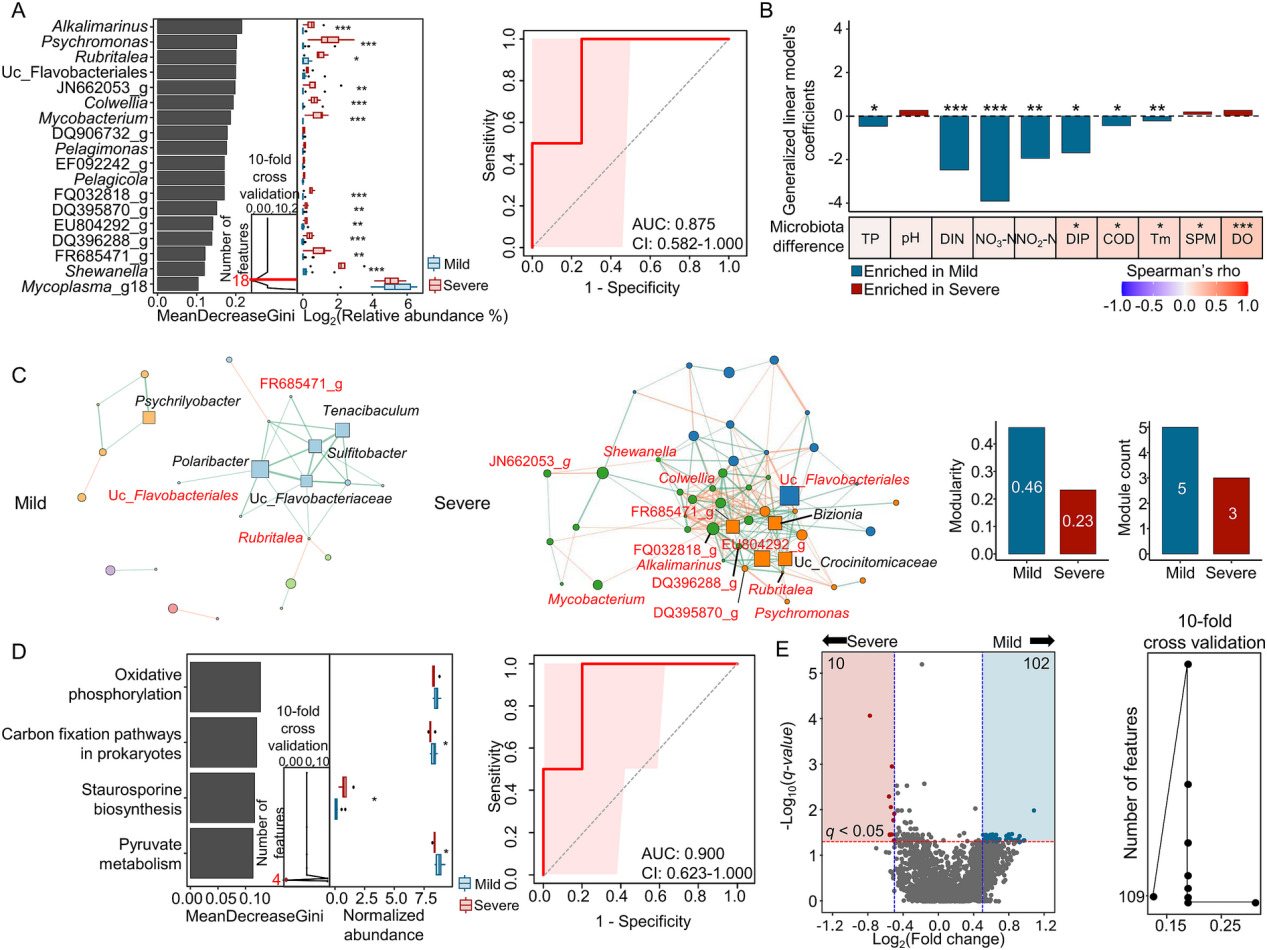
Microbial signatures and interaction structure associated with barren severity in summer. **(A)** Identification of discriminatory genera distinguishing mild and severe barren regions using random forest analysis (summer). The significance of selected genera was evaluated using MaAsLin2, adjusting for sampling site. A refined random forest model was trained on significant features, and its performance was evaluated on a held-out test set (30%) using ROC AUC with 95% confidence intervals (CI). **(B)** Differences in geochemical variables between mild and severe barren regions evaluated by GLMs. Bars represent model coefficients (blue: higher in mild; red: higher in severe). The lower panel displays Spearman correlations between pairwise differences in geochemical variables and Bray–Curtis dissimilarity. **(C)** Co-occurrence networks of gut microbiota in mild and severe barren regions (summer). Correlations were calculated using FastSpar; edges indicate significant associations (*q* < 0.05 and |correlation| > 0.6). Node size reflects PageRank centrality, with the top five nodes identified as keystone taxa. Modularity and module counts were determined using fast-greedy clustering. Discriminatory genera are highlighted in red. **(D)** Random forest identification of discriminatory KEGG pathways (3rd category) between mild and severe barren regions (summer), followed by MaAsLin2 testing with adjustment for sampling site and evaluation of classification performance on the test set (AUC with 95% CI). **(E)** Differential abundance of KO gene families between mild and severe barren regions. The volcano plot illustrates Log_2_ fold changes and –Log_10_ *p*-values (MaAsLin2). Gene families with |log₂ fold change| > 0.5 and *q* < 0.05 were considered significant. The right panel also presents random forest selection of discriminatory KO gene families using 10-fold cross-validation. *q < 0.05, **q < 0.01, ***q < 0.001.

Because barren severity exhibited the clearest microbiota divergence in summer, we further analyzed taxonomic and functional signatures associated with severity within summer samples. Using random forest classification with 10-fold cross-validation, we identified 18 genera as discriminative features that separate mild and severe barren regions (Figure 5A). Of these, 12 genera were significantly more abundant in abalones from the severe region than in those from mild regions (Supplementary Table S11; MaAsLin2 *q* < 0.05) and collectively showed strong classification performance (AUC = 0.875). We defined these 12 taxa as discriminatory genera and examined their environmental associations.

Geochemical parameters differed between mild and severe regions in summer, with TP, DIN, NO_3_-N, NO₂-N, DIP, COD, and Tm significantly lower in the severe region after adjusting for sampling site (GLM *q* < 0.05). In addition, microbiota variation associated with barren severity (Bray–Curtis dissimilarity) was significantly correlated with DIP, COD, Tm, SPM, and DO (Figure 5B; Spearman correlation *q* < 0.05). These results suggest that summer severity-associated shifts in the abalone gut microbiota are linked to coordinated changes in geochemical conditions, likely reflecting altered environmental and dietary statuses across barren regions.

Microbial interactions within the gut microbiota were compared between mild and severe barren regions using a co-occurrence network (Figure 5C). Network analysis revealed differences in highly connected genera between the two groups, with several discriminative genera identified by random forest also occupying central positions in the severe-region network. Although microbial interactions were more complex in the severe region, network modularity and module structure were reduced relative to the mild-region network, indicating reorganization of microbiota connectivity under severe barren conditions.

We next analyzed functional signatures associated with barren severity in summer. Using random forest classification on PICRUSt2-predicted KEGG pathways, we identified four discriminatory pathways via 10-fold cross-validation (Figure 5D), achieving high classification performance (AUC = 0.900). Carbon fixation pathways in prokaryotes and pyruvate metabolism were found to be more abundant in mild than in severe regions (MaAsLin2 *q* = 0.015), whereas staurosporine biosynthesis was more prevalent in the severe region (MaAsLin2 *q* = 0.012). At the gene family level, MaAsLin2 identified 112 gene families differing in abundance between mild and severe regions (10 abundant in severe and 102 abundant in mild; |Log_2_ fold change| > 0.5, *q* < 0.05). Random forest analysis identified 109 discriminatory gene families through 10-fold cross-validation (Figure 5E). Of these, 28 gene families overlapped between the two approaches (Supplementary Table S12), including 20 enriched in mild and eight in severe regions. Together, these taxonomic, network, and functional signatures indicate substantial ecological and functional divergence in the abalone gut microbiota across barren severity during summer.

## 4 Discussion

This study analyzed the gut microbiota of abalones collected in winter and summer from four barren regions and one aquaculture site in South Korea. Overall, the results show that abalone gut microbiota varied according to life type (wild vs. farmed), season, and barren severity, with the clearest severity-associated divergence occurring in summer. These variations were closely associated with site- and season-dependent geochemical variation, together with likely differences in dietary resource availability across habitats.

The gut microbiota of wild abalone differed from that of farmed abalone, reflecting the contrasting habitat conditions and feeding regimes of natural reefs and aquaculture systems. Distinct sets of environmental variables were associated with microbiota variation in the two groups, suggesting that the dominant ecological constraints differed between habitats. In farmed abalone, DO, COD, TP, and SPM were significantly associated with gut microbiota variation, whereas NO_2_-N, NO_3_-N, DIN, and pH were associated with the gut microbiota of wild abalone. Previous studies have highlighted the influence of DO, COD, TP, and SPM in abalone aquaculture systems (Harris et al., 1999; Chen et al., 2020; Sim et al., 2021). Among these farm-associated variables, SPM showed the highest coefficient and the broadest associations with the genera that differentiated wild and farmed abalone. SPM can act as a microhabitat and dispersal vectors for diverse microorganisms (Kramer et al., 2016), and particle-attached microbes can contribute substantially to the processing of algal-derived substrates (Wang et al., 2024). However, the significant associations of DO, COD, and TP indicate that microbiota divergence in farmed abalones cannot be explained by particulate conditions alone. DO is particularly relevant from a host-physiological perspective because reduced oxygen availability can directly constrain abalone respiration and growth, and temporal monitoring of *Haliotis discus hannai* has linked intestinal bacterial community shifts to DO and related water-quality variables (Harris et al., 1999; Zhang et al., 2022). COD and TP are commonly interpreted as indictors of organic and nutrient loading in aquaculture systems, where excess feed and waste can increase oxygen demand and phosphorus accumulation (Zhang et al., 2024; Li et al., 2025a). Collectively, these results suggest that the gut microbiota of farmed abalone is associated with a broader suite of particulate, oxygen, and nutrient conditions, with SPM representing the most pervasive correlation in the present study rather than the sole driver. By contrast, variations in nitrogen compounds (NO_2_, NO_3_, and DIN) may influence microbiota by altering nutrient availability and redox conditions, thereby favoring microbes with different nitrogen utilization strategies (Dang and Chen, 2017; Zhu et al., 2020). Environmental pH can further modulate microbial energetics and community assembly and has been reported to correlate with abalone gut microbiota (Zhang et al., 2022). Taken together, these findings support the notion that differences in gut microbiota between wild and farmed abalones are associated with habitat-specific variations in particulate exposure, oxygen status, and nutrient chemistry.

The Shannon diversity patterns provide additional ecological context for these habitat-associated differences. Because Shannon diversity reflects both richness and evenness, it was used here to interpret overall microbiota structuring rather than richness alone. By contrast, richness estimators such as Chao1 are more strongly influenced by rare taxa, and we considered them less suitable for the present dataset because the workflow included decontamination and prevalence with abundance filtering to reduce the influence of rare and potentially unstable features in low-biomass samples. Higher diversity should not be interpreted as a more beneficial gut microbiota state. In Pacific abalone, higher intestinal microbial diversity has been reported in slower-growing individuals (Choi et al., 2021). In the present study, farmed abalones showed higher diversity than wild abalones, particularly in summer, despite no significant difference in total bacterial load. Although not directly tested here, this may reflect aquaculture-specific conditions, in which regular feed input and waste accumulation increase particulate and nutrient loading, thereby enhancing exposure to particle-associated microbial assemblages (Lee et al., 2016; Wang X. Y. et al., 2020). Because marine particles are hotspots of microbial colonization (Baumas and Bizic, 2024) and abalone gut microbiota is responsive to feeding pattern, the higher summer diversity in farmed abalones may indicate community broadening or greater evenness rather than a simple increase in bacterial biomass (Guo et al., 2024). By contrast, the higher diversity but lower bacterial load observed in severe barren regions during summer may reflect a decline in dominant diet-associated taxa under macroalgal limitation together with a relative increase in alternative low-abundance taxa. This is consistent with previous abalone studies showing a persistent core microbiota overlaid by a diet-responsive minor component, as well as degraded-habitat studies in marine animals reporting increased gut microbiome diversity and variability under altered feeding or environmental stress (Gobet et al., 2018; Clever et al., 2022). Overall, the higher diversity observed here likely reflects community restructuring under different ecological conditions rather than as an intrinsically positive or negative outcome for host health.

Seasonal variations in gut microbiota were detected in both wild and farmed abalone. In wild abalone, DO, TP, NO_2_-N, and COD were significantly associated with seasonal microbiota differences, whereas Tm showed only a weak association and was not significant after FDR correction. Although Tm is often considered a primary seasonal driver because microbial growth and alterations in microbiota are temperature-sensitive, and Tm can also related to broader geochemical factors (Song et al., 2019; Li et al., 2025b), our results suggest that seasonal microbiota shifts were more closely associated with temperature-related geochemical variation than with temperature alone. Farmed abalone also exhibited seasonal microbiota shifts, but inference about environmental drivers was limited because samples were obtained from a single location and geochemical measurements were not replicated at the individual sample level within each season. Nonetheless, the observed seasonal changes in both geochemical variables and microbiota are consistent with the idea that seasonal gut microbiota turnover is associated with broader environmental change.

In wild abalone, seasonal microbiota differences were closely associated with COD and DO. The positive correlation between COD and the higher abundance of *Vibrio* during summer suggests that seasonally enhanced organic loading may favor *Vibrio* under warm coastal conditions. This is consistent with previous studies showing that *Vibrio* abundance in Korean coastal waters increases during summer, together with year-rounding monitoring in aquaculture-associated bays showing that the highest *Vibrio* abundance in summer (Lee et al., 2019; Kim and Chun, 2021; Kim et al., 2024). However, because pollution levels were not directly measured at our sampling sites, this pattern should not be interpreted as direct evidence of site-specific pollution. Rather, it should be viewed more conservatively as being compatible with warm, organic-enriched coastal conditions, while acknowledging that unmeasured regional pollution may also have contributed to the observed relationship. By contrast, the negative association between DO and the higher abundance of *Propionigenium* aligns with the decrease in DO under warmer conditions (Song et al., 2019) and with the host’s reliance on anaerobic metabolism when oxygen availability is low (Venter et al., 2018). Elevated Tm can increase metabolic demand and feeding activity in marine gastropods (Foster and Hodgson, 1998; Kang et al., 2019), suggesting that the enrichment of strict anaerobes such as *Propionigenium*, which can ferment succinate to propionate (Hakim et al., 2019), may reflect gut microbiome responses associated with summer metabolic conditions.

The effects of barren severity were clearly season-dependent. In winter, neither microbial diversity nor bacterial loads showed significant differences after FDR correction, and differences in gut microbiota between mild and severe regions were not evident. By contrast, the divergence between mild and severe barren regions was more pronounced in summer than variation among mild sites. The significant correlation between algal community composition and microbiota differences suggests that dietary factors influence microbiota variation in summer. Higher seawater temperatures increase metabolic demands and food consumption (Kang et al., 2019; Seo and Koo, 2023), whereas lower temperatures suppress feeding activity and gut turnover (Alcantara and Noro, 2005), potentially dampening diet-linked microbiota differences in winter. Consequently, summer appears to be the period during which dietary restrictions associated with barren severity are most likely to influence the gut microbiota.

After adjusting for sampling site, we found a significant summer-specific divergence in gut microbiota between mild and severe barren regions at both taxonomic and predicted functional levels. Although phylum-level differences provided a general overview of microbiota restructuring, the ecological interpretation is better supported by lower taxonomic patterns, particularly the discriminatory genera identified in summer and their associated functional shifts. Carbohydrate metabolism was higher in mild regions, likely due to increased availability of macroalgae as a dietary substrate for abalone (Pan et al., 2018). By contrast, the severe barren region, characterized by reduced macroalgal biomass, exhibited distinct microbial signature consistent with nutritional constraints. Geochemical variables, including DIP, COD, and Tm also differed across severity levels and were correlated with microbiota differences, suggesting that dietary limitation related to barren severity and associated geochemical differences together contribute to gut microbiota divergence. More broadly, the co-occurrence of nutrient depletion, organic loading, and temperature-associated stress has been linked to kelp decline and hindered recovery potential in barren systems (Paytan and McLaughlin, 2007; Krumhansl et al., 2016; Kemena et al., 2019; Filbee-Dexter et al., 2020). Because abalone physiology is also highly Tm-dependent, responding not only to seasonal variations but also to short-term thermal fluctuations (Kang et al., 2019), such environmental heterogeneities may help explain the clear summer divergence in gut microbiota associated with barren severity.

Functionally, severe barren regions showed reduced predicted abundance of key energy-related pathways, including carbon fixation pathways in prokaryotes and pyruvate metabolism, suggesting lower microbial energy metabolism consistent with diminished dietary energy flow to the gut. At the gene-family level, these pathway-level differences were supported by lower abundance of K00175 (related to pyruvate metabolism and carbon fixation in prokaryotes) indicating reduced central energy production and respiratory metabolism under severe barren conditions. By contrast, K00138 (aldehyde dehydrogenase) was more abundant in severe regions. Given that aldehyde dehydrogenases are typically upregulated under environmental stress, including oxidative stress, and contribute to detoxification of reactive aldehydes (Singh et al., 2013), this pattern may reflect microbial stress adaptation rather than enhanced caron flux. Because food limitation can increase oxidative stress responses in marine invertebrates (Hossen et al., 2023; Duarte et al., 2025), enrichment of K00138 in severe barren regions may suggest a gut environment more closely associated with stress. Nonetheless, these interpretations remain predictive and require validation using shotgun metagenomics and targeted metabolite measurements.

Another notable predicted functional signal associated with severity was enrichment of the staurosporine biosynthesis pathway in the abalone gut microbiome. This may indicate a shift toward secondary metabolism and microbial competition under nutrient-limited conditions. Staurosporine is a potent, broad-spectrum protein kinase inhibitor (Tamaoki et al., 1986) widely used to induce apoptosis in eukaryotic cells (Feng and Kaplowitz, 2002). If this biosynthetic potential correspond to increased intraluminal exposure, it could potentially affect the host epithelial physiology, because apoptosis in epithelial cells is associated with redistribution of tight-junction proteins and compromised barrier integrity (Bojarski et al., 2004). However, this interpretation remains speculative in the absence of direct metabolite quantifications and should be viewed as testable hypothesis rather than a demonstrated mechanism.

This study has several limitations. First, functional profiles were predicted using PICRUSt2 based on 16S rRNA genes rather than being directly measured through shotgun metagenomics or metabolomics, which limits mechanistic interpretation of pathway differences. Second, although we adjusted for sampling site in our comparisons, environmental variation and dietary composition are challenging to disentangle in field settings. This complexity may also explain the relatively low R^2^ values of individual environmental variables, because each measured factor is likely to explain only part of the total microbiota variation in a complex natural system. Similar modest explained variance has been reported in both large gut microbiome cohort studies and host-associated marine microbiome studies (Zhernakova et al., 2016; Gobet et al., 2018; Lokmer et al., 2020), supporting the interpretation that biologically meaningful associations may still occur despite limited effect sizes of single variables. In addition, algal availability was assessed at the community level rather than through direct quantification of individual diets. Third, farmed abalones were sourced from a single location, limiting the generalizability of comparisons between wild and farmed specimens and restricting inference about farm-specific environmental drivers. Fourth, another host-related factor that may have contributed to unexplained microbiota variation is sex and reproductive status. Although these were not evaluated in the present field survey, increasing evidence suggests that host sex can influence gut microbiota in aquatic animals, and broader studies of the gut microbiota-gonadal axis indicate that endocrine state may also shape microbial community composition and function (Bates et al., 2023; Ashonibare et al., 2024). Future studies should therefore incorporate host sex and gonadal stage to determine whether part of the seasonal or habitat-associated variation observed here is linked to sex-specific physiology. Future research integrating shotgun metagenomics, metabolomics, and controlled feeding experiments will be necessary to validate the ecological and functional patterns identified here.

## 5 Conclusion

Our findings suggest that the gut microbiota of abalones is associated with seasonal variation and geochemical factors that differ according to barren severity, with the strongest microbiota divergence observed during summer. Notably, the effects of barren severity were most pronounced under warm conditions, consistent with increased feeding activity and stronger dietary influence on gut microbiota during summer. In severe barren regions, changes in microbe–microbe interactions and distinct predicted functional signatures were observed, including enrichment of secondary metabolism (specifically staurosporine biosynthesis) and depletion of core energy-related pathways. Together, these patterns suggest that dietary limitations and geochemical variation are associated with restructuring of the gut microbial ecosystem and may be linked to differences in microbiome-related aspects of host physiology. These results provide ecological insights into how geochemical variations and resource limitations in barren regions are associated tih differences in abalone gut microbiota. Further studies are needed to determine whether and how these microbiota differences influence host resilience across varying degrees of barrenness.

## Data Availability

The datasets presented in this study can be found in online repositories. The names of the repository/repositories and accession number(s) can be found at: https://www.ebi.ac.uk/ena, PRJEB89929.
