# Supplementary material for: Geochemical factors associated with seasonal shifts in the abalone gut microbiota across urchin-barren habitats
Source: Front Microbiol. 2026 May 8;17:1817344. doi: 10.3389/fmicb.2026.1817344 (PMC13194499; doi:10.3389/fmicb.2026.1817344)
Supplement: Supplementary file 1 [file Data_Sheet_1.PDF]

Supplementary materials

Geochemical factors associated with seasonal shifts in the abalone gut microbiota across urchin-barren habitats

Jae-Won Jo[1], Joon-Young Park[1], Jin-Jae Lee[1], Min-Jung Lee[2], Bong-Soo Kim[2,3]*

[1]Department of Life Science, Multidisciplinary Genome Institute, Hallym University, Chuncheon, Gangwon-do 24252, Republic of Korea

[2]Department of Nutritional Science and Food Management, Ewha Womans University, Seoul 03760, Republic of Korea

[3]Global Food and Nutrition Research Institute, Ewha Womans University, Seoul 03760, Republic of Korea

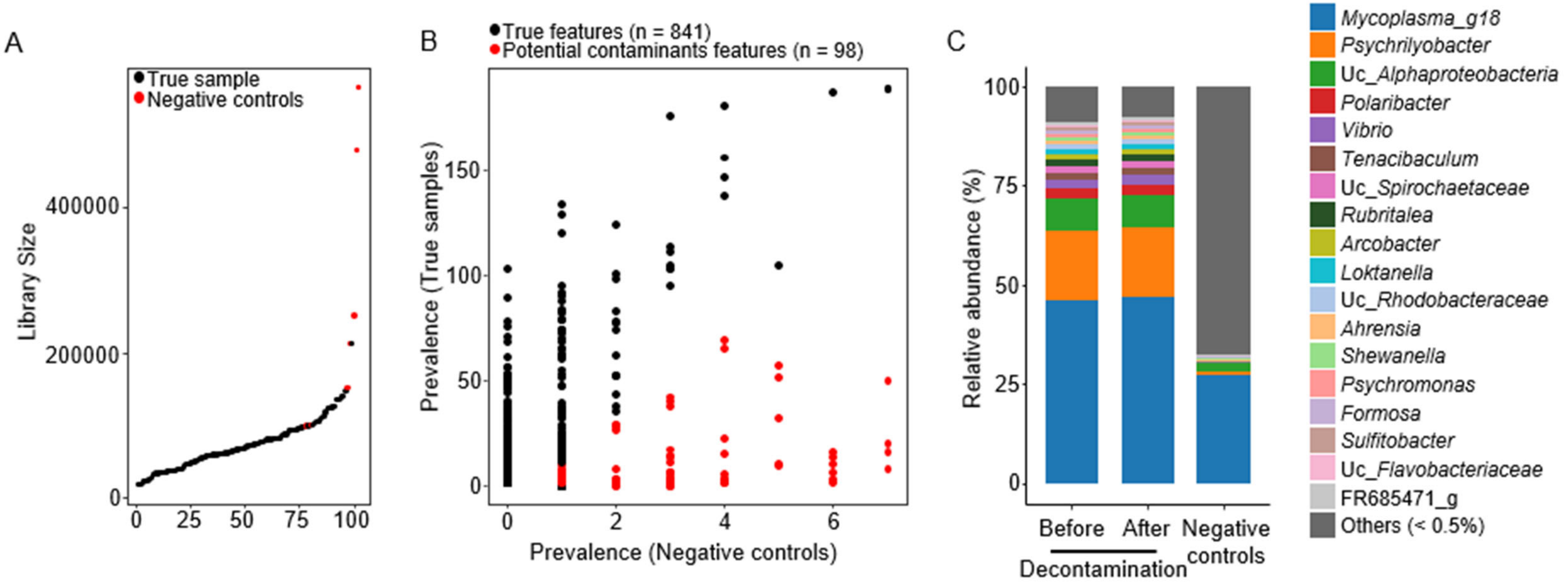

Fig. S1. Decontamination based on sequences from negative controls. (A) Library size for true samples (n = 95) and negative control samples (n = 7). (B) Prevalence (%) of putative contaminants (red) and non-contaminants (black) identified by the prevalence-based method, plotted for true samples (*y* axis) and negative controls (*x* axis). (C) Genus-level relative abundance in true samples before and after decontamination as well as in negative controls. Genera with a relative abundance of < 0.5% across all samples were categorized as "Others".

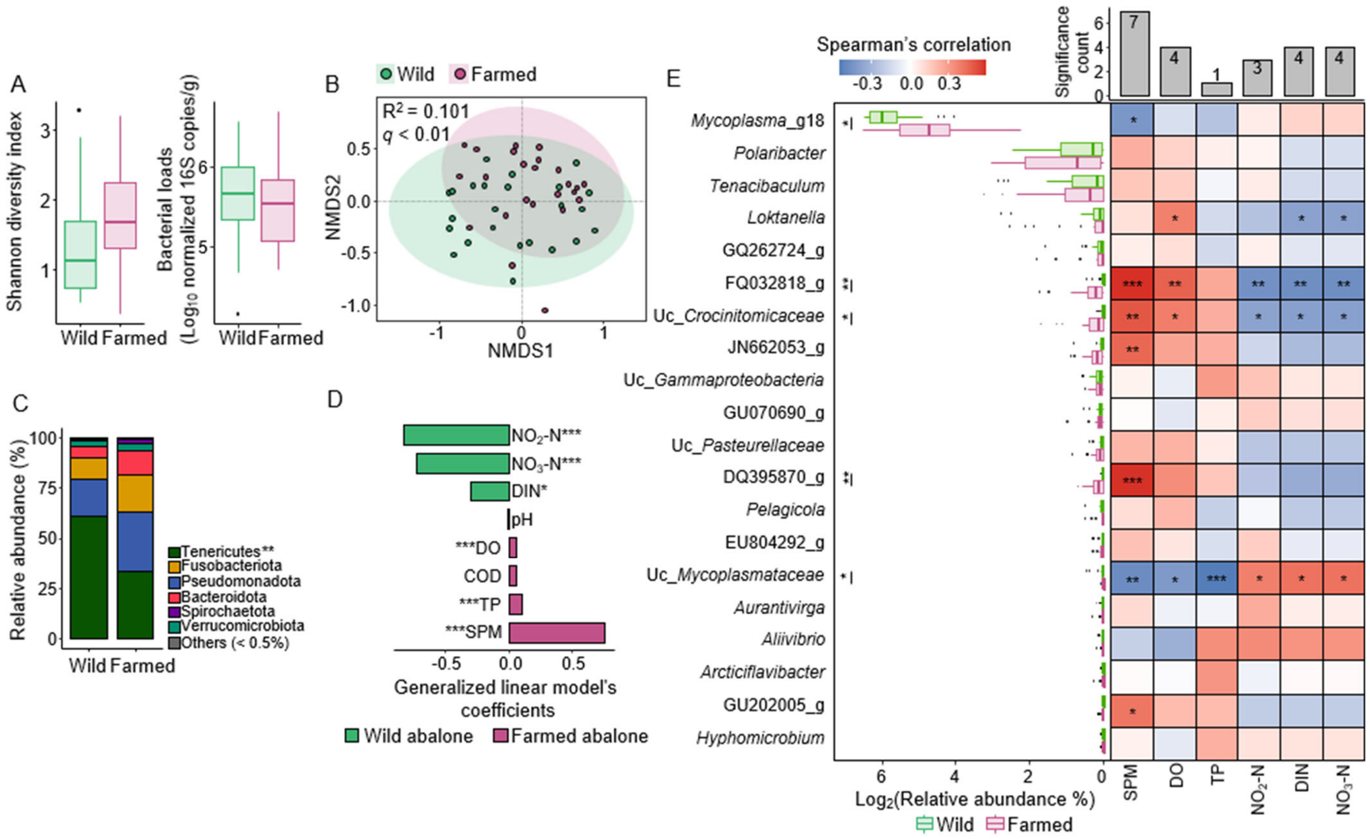

Fig. S2. Differences in gut microbiota between wild and farmed abalones in winter. (A) Shannon diversity and bacterial load ($\log_{10}$ 16S rRNA gene copies/g) in wild (n = 24) and farmed (n = 24) abalones. Differences were tested using GLM. (B) NMDS ordination based on Bray–Curtis dissimilarity showing differences in gut microbiota composition between wild and farmed abalones; significance was assessed by PERMANOVA. (C) Relative abundance of dominant phyla in wild and farmed abalones. Phyla with a relative abundance of < 0.5% across all samples were categorized as "Others". Differences were tested using the Wilcoxon rank-sum test. (D) Differences in geochemical factors between wild and farmed environments estimated by GLMs. Negative and positive coefficients indicate higher levels in wild and farmed environments, respectively. (E) Heatmap showing associations between 20 genera that showed nominal differences between wild and farmed abalones (Wilcoxon rank-sum test, $p < 0.05$), five of which remained significant after FDR correction ($q < 0.05$), and geochemical factors that also differed between the two environments. All 20 nominally significant genera were included in the association analysis, and the bar plot indicates the number of genera significantly associated with each geochemical factor (Spearman's correlation). *$q < 0.05$, **$q < 0.01$, ***$q < 0.001$.

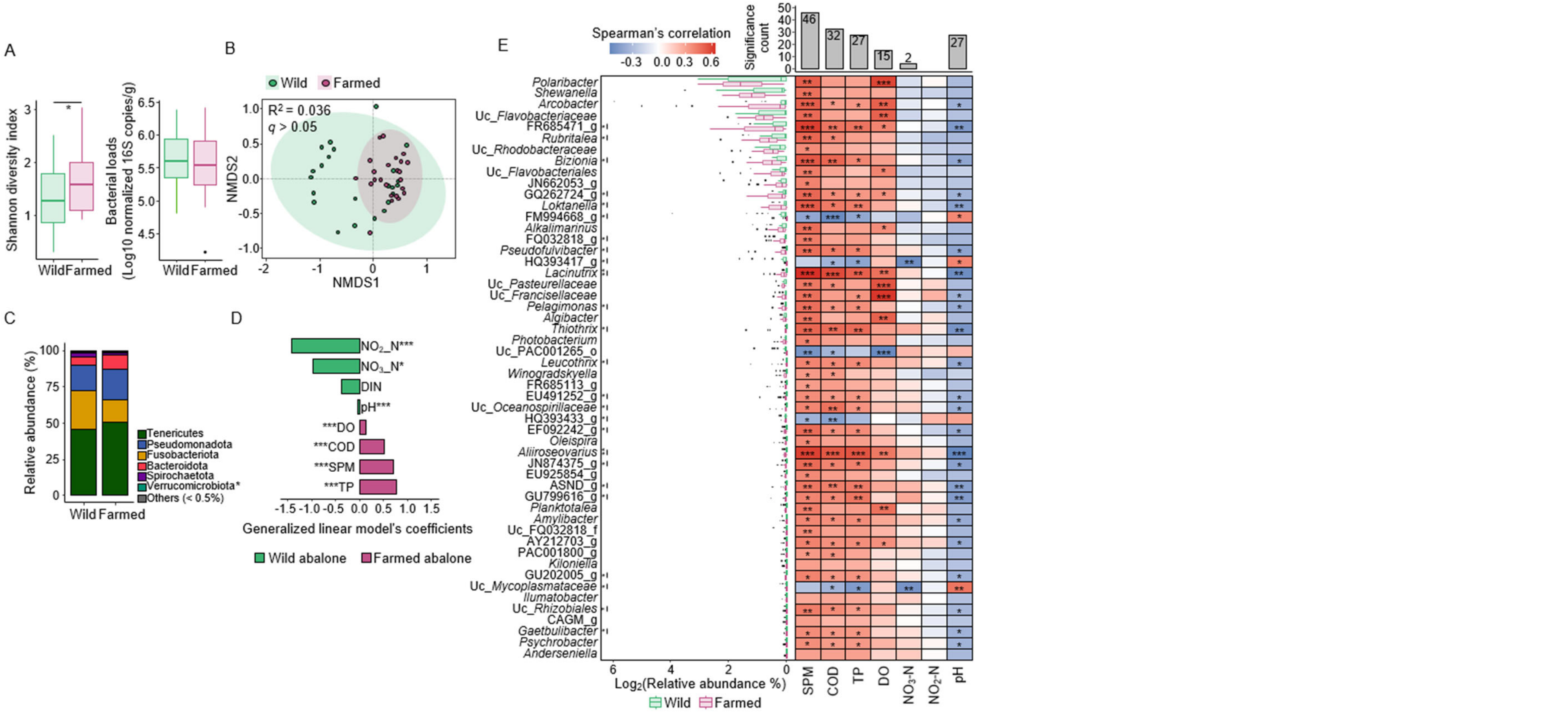

Fig. S3. Differences in gut microbiota between wild and farmed abalones in summer. (A) Shannon diversity and bacterial load (log_{10} 16S rRNA gene copies/g) in wild (n = 23) and farmed (n = 24) abalones. Differences were tested using GLMs. (B) NMDS ordination based on Bray–Curtis dissimilarity illustrating the differences in gut microbiota composition between wild and farmed abalones, with significance assessed by PERMANOVA. (C) Relative abundance of dominant phyla in wild and farmed abalones, where phyla with < 0.5% relative abundance across all samples are categorized as "Others". Differences were evaluated using the Wilcoxon rank-sum test. (D) Geochemical factors differing between wild and farmed environments estimated by GLMs, with negative and positive coefficients indicating higher levels in wild and farmed environments, respectively. (E) Heatmap showing associations between 52 genera that showed nominal differences between wild and farmed abalones (Wilcoxon rank-sum test, $p < 0.05$), 25 of which remained significant after FDR correction ($q < 0.05$), and geochemical factors that also differed between the two environments. All 52 nominally significant genera were included in the association analysis, and the bar plot indicates the number of genera significantly associated with each geochemical factor (Spearman's correlation). $*q < 0.05$, $**q < 0.01$, $***q < 0.001$.

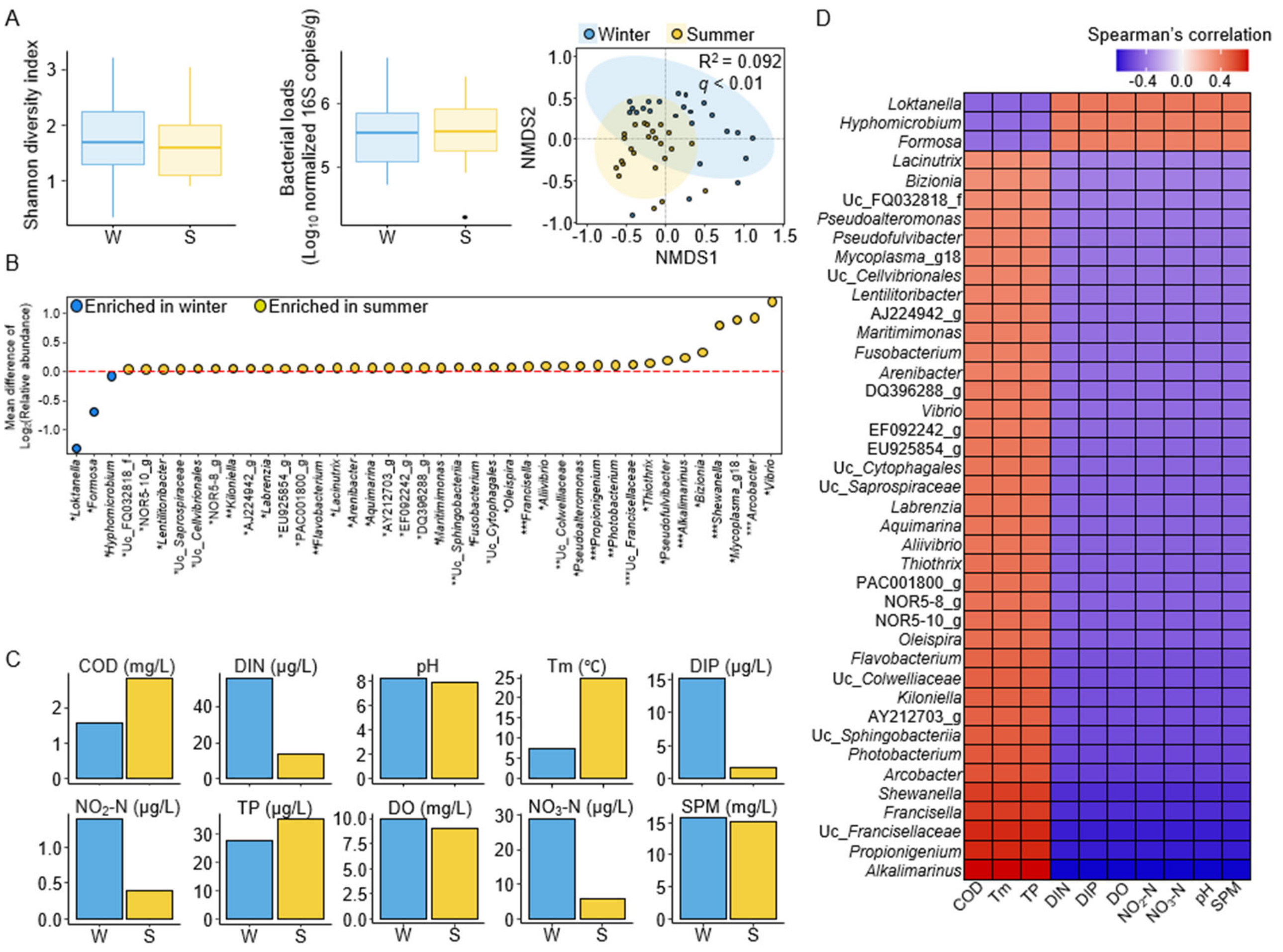

Fig. S4. Seasonal variation of gut microbiota in farmed abalones. (A) Shannon diversity and bacterial load (log_{10} 16S rRNA gene copies/g) in winter versus summer, and NMDS ordinations based on Bray–Curtis dissimilarity for gut microbiota between seasons. (B) Differentially abundant genera identified between winter and summer using the Wilcoxon rank-sum test ($q < 0.05$). (C) Seasonal profiles of 10 geochemical factors. (D) Spearman correlation analysis showing associations between 10 geochemical factors and 63 genera exhibiting significant seasonal differences, with genera ordered by the difference in mean log_2(relative abundance) between seasons.

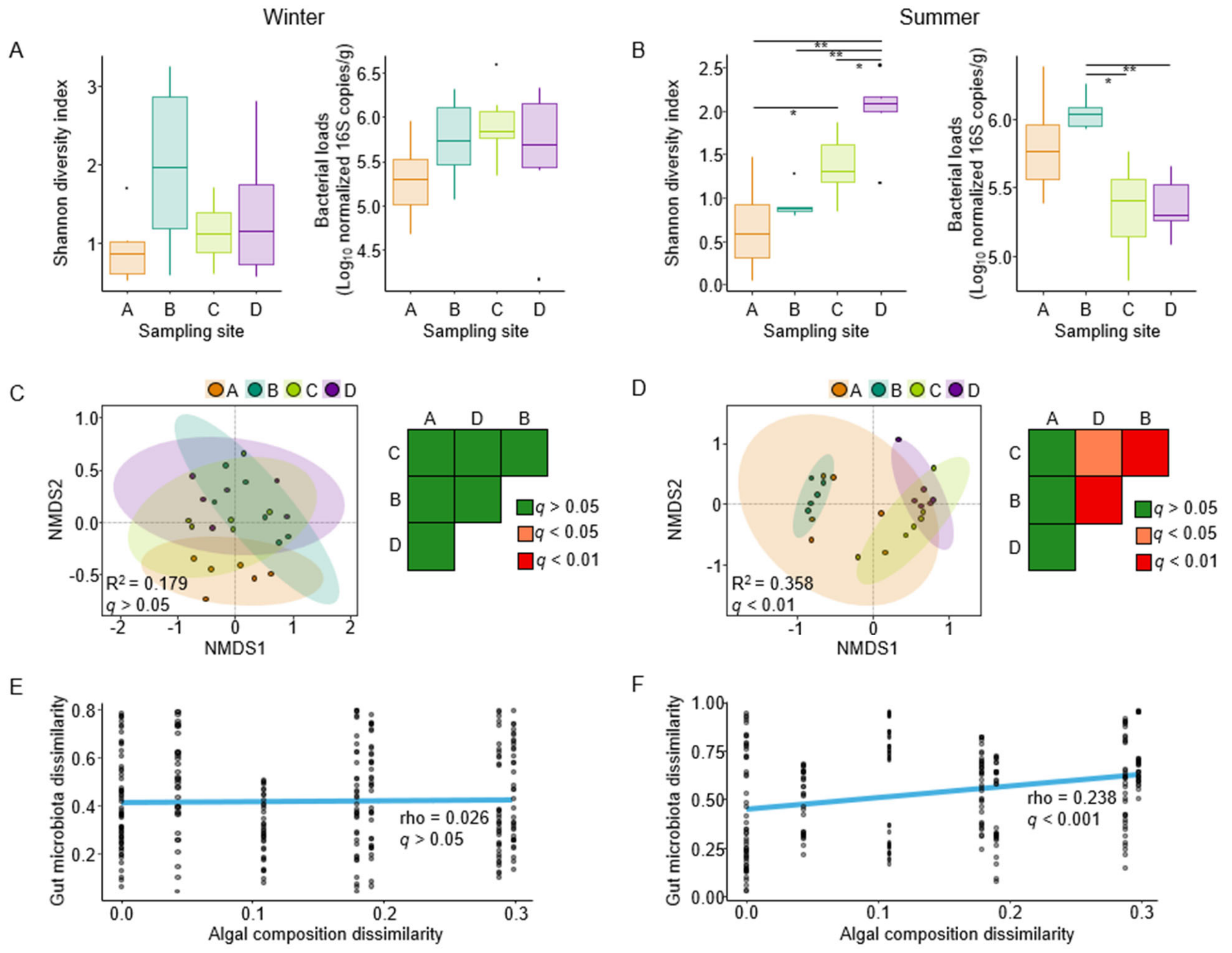

Fig. S5. Site-level variation in gut microbiota and algal composition within each season. Shannon diversity and bacterial load ($\log_{10}$ 16S rRNA gene copies/g) for sampling sites in winter (A) and summer (B); significance was evaluated using GLMs. NMDS ordination based on Bray–Curtis dissimilarity showing gut microbiota composition across sampling sites in winter (C) and summer (D); significance was assessed using PERMANOVA. The heatmap displays pairwise comparisons between sites. Spearman correlation between Bray–Curtis dissimilarity of gut microbiota and algal composition dissimilarity across sites in winter (E) and summer (F). All statistical significance shown in this figure was determined based on FDR-corrected q-values. *$q < 0.05$, **$q < 0.01$, ***$q < 0.001$.

Table S1. Geochemical characteristics of sampling sites and abalone samples collected.

Characteristic		Sampling month									
		February					August				
Sampling	Site	A	B	C	D	E	A	B	C	D	E
	Region	Goseong	Tongyeong	Yeosu	Pohang	Wando	Goseong	Tongyeong	Yeosu	Pohang	Wando
	Abalone category (Wild/Farmed)	Wild	Wild	Wild	Wild	Farmed	Wild	Wild	Wild	Wild	Farmed
	The number of samples (n)	6	6	6	6	24	6	5	6	6	24
Geochemical information	Seawater temperature (℃)	4.4	10.05	6.6	11.48	7.57	26.93	20.3	24.67	19.95	24.73
	Salinity (PSU[a])	33.98	34.12	33.42	34.29	33.26	29.04	33.44	32.15	31.29	31.87
	pH	8.13	8.24	8.34	7.9	8.14	8.32	8.09	8.15	8.21	7.86
	Dissolved oxgen (㎎/L)	9.19	9.38	10.46	8.67	10	7.55	6.27	9.7	8.21	9.06
	Chemical oxygen demand (㎎/L)	1.78	1.34	2.23	0.51	1.56	2.55	0.92	1.98	1.2	2.82
	Ammonium-nitrogen (μg/L)	11.1	8.3	10.3	16.6	25.1	1.8	3.6	7.3	1.5	7.6
	Nitrite-nitrogen (μg/L)	2.4	4.9	1.1	4.5	1.4	0.1	6.5	0.5	0.3	0.4
	Nitrate-nitrogen(μg/L)	73.7	66.8	1.5	98.5	29	0.9	63.9	2.6	0.4	5.7
	Dissolved inorganic nitrogen (μg/L)	87.2	80	13	119.7	55.5	2.8	74	10.3	2.2	13.7
	Dissolved inorganic phosphorus (μg/L)	13.3	14	3.1	16	15.1	0.4	12.2	0.2	0.7	1.7
	Total nitrogen (μg/L)	169.8	162.2	141.8	189.6	140.6	130.5	226.6	176.9	120.1	231.9
	Total phosphorus (μg/L)	22.9	24.4	24.8	27.8	27.7	8.6	27.4	19.8	11.3	35.2
	Silicic acid (μg/L)	158.8	318.2	49.3	313.6	321	41.4	481.3	67.3	86.6	375.3
	Chlorophyll A (μg/L)	0.59	1.49	2.74	0.2	0.53	0.92	1.04	0.39	0.73	0.72
	Suspended particulate matter (㎎/L)	3.1	11.9	12.6	1.6	15.7	7.7	5.3	8.6	8	15.2
Severity information	Barrens severity	Mild	Mild	Mild	Severe	-	Mild	Mild	Mild	Severe	-
	Normal area (%)	53.1	84.2	84.9	40.7	-	53.1	84.2	84.9	40.7	-

Mild area (%)	31.4	8.5	8.9	34.2	-	31.4	8.5	8.9	34.2	-
Severe area (%)	15.5	7.4	6.2	25.1	-	15.5	7.4	6.2	25.1	-

[a]PSU: Practical salinity unit

Table S2. Summary of urchin barren survey reports along the South Korean coast, provided by the Korea Fisheries Resources Agency (FIRA; https://www.fira.or.kr), collected in 2019.

Coast	Site	Survey area (ha)	Barren area					
			Area (ha)			Ratio (%)		
			Normal	Mild	Severe	Normal	Mild	Severe
Eastern	Gosung	358.1	190.4	112.3	55.4	53.1	31.4	15.5
	Sokcho	358.1	190.4	112.3	55.4	53.1	31.4	15.5
	Yangyang	468.2	270.1	112.0	86.1	57.7	23.9	18.4
	Gangneung	853.0	514.2	190.2	148.6	60.3	22.3	17.4
	Donghae	638.4	369.8	180.1	88.5	57.9	28.2	13.9
	Samcheok	652.2	404.2	153.3	94.7	62.0	23.5	14.5
	Ulleung do	794.7	650.6	94.6	49.5	81.9	11.9	6.2
	Uljin	1,004.2	494.9	248.5	260.8	49.3	24.7	26.0
	Yeongdeok	789.3	378.4	182.9	228.0	47.9	23.2	28.9
	Pohang	4,263.1	1,734.0	1,457.0	1,072.1	40.7	34.2	25.1
	Gyeongju	773.2	414.6	207.2	151.4	53.6	26.8	19.6
	Ulsan	1,686.7	825.9	446.0	414.8	49.0	26.4	24.6
	Busan	1,144.0	695.2	279.3	169.5	60.8	24.4	14.8
Southern	Changwon	122.0	106.7	10.9	4.4	87.5	8.9	3.6
	Geoje	726.6	628.3	63.7	34.6	86.5	8.8	4.8
	Tongyeong	1,203.4	1,012.9	101.9	88.6	84.2	8.5	7.4
	Sacheon	165.3	155.8	6.0	3.5	94.3	3.6	2.1
	Gwangyang	0.5	0.5	0.0	0.0	100.0	0.0	0.0
	Namhe	558.0	458.9	85.0	14.1	82.2	15.2	2.5
	Hadong	47.3	43.3	3.5	0.5	91.5	7.4	1.1
	Yeosu	1,948.3	1,654.9	173.3	120.1	84.9	8.9	6.2
	Suncheon	1.2	1.2	0.0	0.0	100.0	0.0	0.0
	Boseong	31.8	29.5	2.1	0.2	92.8	6.6	0.6
	Goheung	915.6	861.0	36.1	18.5	94.0	3.9	2.0
	Jangheung	44.6	40.9	1.7	2.0	91.7	3.8	4.5
	Gangjin	11.4	10.4	0.8	0.2	91.2	7.0	1.8
	Haenam	179.9	161.4	11.6	6.9	89.7	6.5	3.8

Table S3. Regional abalone production (tons) in the Republic of Korea for February and August (2020–2022). Data sourced from the Korean Statistical Information Service (https://kosis.kr/publication/publicationThema.do), under the Agriculture/Forestry and Fisheries section, within the Fisheries Production Trend Survey tab.

Administrative Regions	Annual reported production of abalone (tons)					
	2020		2021		2022	
	February	August	February	August	February	August
Busan	1	2	2	1	1	1
Ulsan	2	2	6	1	1	1
Gangwon-do	0	0	0	0	0	2
Chungcheongnam-do	0	0	2	1	0	2
Jeollanam-do	1,048	1,634	1,345	1,924	1,183	2,683
Gyeongsangbuk-do	0	8	3	0	1	3
Gyeongsangnam-do	8	10	8	3	12	4
Jeju-do	2	23	2	2	1	2

Table S4. Number and proportion of farmed abalone aquaculture units by region in South Korea. Data obtained from the Abalone Aquaculture Management Manual for Aquaculture Farmers (2019), published by the National Institute of Fisheries Science (NIFS), South Korea (https://www.nifs.go.kr/cmmn/file/farm/farm_12.pdf).

Site	Number of units	Proportion (%)
Taean	3,239	0.32
Shinan	61,529	6.16
Jindo	106,759	10.69
Haenam	75,447	7.55
Wando	728,908	72.99
Jangheung	150	0.02
Goheung	14,297	1.43
Yeosu	3,324	0.33
Tongyeong	4,332	0.43
Geoje	715	0.07

Table S5. Significantly different gut microbiota genera between wild and farmed abalones after FDR correction.

Genus	*p-value*	*q-value*
Loktanella	0.000006	0.000217
FQ032818_g	0.000010	0.000275
DQ395870_g	0.000036	0.000681
Tenacibaculum	0.000105	0.001681
Polaribacter	0.000205	0.002741
GQ262724_g	0.000236	0.002741
JN662053_g	0.000424	0.004169
FR685471_g	0.000447	0.004169
Uc_Crocinitomicaceae	0.000764	0.006108
Uc_Pasteurellaceae	0.001020	0.006742
Rubritalea	0.001023	0.006742
Shewanella	0.002017	0.012551
Bizionia	0.003613	0.021297
Pseudofulvibacter	0.004604	0.025785
Uc_Rhodobacteraceae	0.005299	0.028259
Uc_Flavobacteriales	0.006224	0.031685
Pelagicola	0.007810	0.037512
GU070690_g	0.008038	0.037512
Formosa	0.008621	0.037734
Alkalimarinus	0.008760	0.037734
Uc_Gammaproteobacteria	0.012135	0.048540

Table S6. Significantly different gut microbiota genera between wild and farmed abalones in winter after FDR correction.

Genus	*p-value*	*q-value*
DQ395870_g	0.000113	0.009436
FQ032818_g	0.000136	0.009436
*Mycoplasma*_g18	0.000661	0.03061
Uc_*Crocinitomicaceae*	0.001075	0.037349
Uc_*Mycoplasmataceae*	0.001376	0.038255

Table S7. Significantly different gut microbiota genera between wild and farmed abalones in summer after FDR correction.

Genus	*p-value*	*q-value*
Lacinutrix	7.92E-05	0.007922
Aliiroseovarius	0.000112	0.007922
FR685471_g	0.00045	0.018583
Bizionia	0.000767	0.018583
Loktanella	0.000785	0.018583
Thiothrix	0.000639	0.018583
FM994668_g	0.001497	0.02823
Pseudofulvibacter	0.001689	0.02823
JN874375_g	0.001882	0.02823
GU799616_g	0.001988	0.02823
EF092242_g	0.002251	0.029063
ASND_g	0.002484	0.029391
Uc_Mycoplasmataceae	0.002805	0.030644
Uc_Oceanospirillaceae	0.003507	0.035572
Rubritalea	0.004144	0.036777
Pelagimonas	0.003896	0.036777
HQ393433_g	0.004883	0.040789
HQ393417_g	0.005499	0.043383
GQ262724_g	0.006205	0.046371
FQ032818_g	0.008586	0.049291
Leucothrix	0.007125	0.049291
EU491252_g	0.008678	0.049291
GU202005_g	0.007849	0.049291
Uc_Rhizobiales	0.00858	0.049291
Gaetbulibacter	0.008678	0.049291

Table S8. Significantly different gut microbiota genera in wild abalones between winter and summer.

Genus	*p-value*	*q-value*
Propionigenium	3.50E-07	8.53E-05
Loktanella	0.00193	0.024271
Uc_Crocinitomicaceae	0.007109	0.050311
Uc_Bacillales	0.011654	0.072912
Formosa	0.01794	0.09096
Vibrio	0.024467	0.106097
Alkalimarinus	0.024785	0.106097
JN874375_g	0.028028	0.113979
*Mycoplasma*_g18	0.033119	0.128269
HQ393417_g	0.033721	0.128561
Lacinutrix	0.047601	0.170803

Table S9. Significantly different gut microbiota genera in farmed abalones between winter and summer.

Genus	*p-value*	*q-value*
Alkalimarinus	0.000002	0.000229
Propionigenium	0.000007	0.000352
Uc_Francisellaceae	0.000008	0.000352
Shewanella	0.000010	0.000352
Francisella	0.000026	0.000733
Arcobacter	0.000164	0.003911
Photobacterium	0.000247	0.005046
Uc_Sphingobacteriia	0.000338	0.006034
Uc_Colwelliaceae	0.000519	0.006742
AY212703_g	0.000519	0.006742
Kiloniella	0.000519	0.006742
Flavobacterium	0.000700	0.008346
Oleispira	0.001388	0.015271
NOR5-10_g	0.001511	0.015437
NOR5-8_g	0.001939	0.016465
PAC001800_g	0.001971	0.016465
Thiothrix	0.001972	0.016465
Aliivibrio	0.002377	0.016465
Aquimarina	0.002418	0.016465
Labrenzia	0.002418	0.016465
Uc_Saprospiraceae	0.002418	0.016465
Uc_Cytophagales	0.002608	0.016952
EU925854_g	0.002790	0.017344
Loktanella	0.003071	0.018299
EF092242_g	0.003853	0.021403
Vibrio	0.003891	0.021403
DQ396288_g	0.004054	0.021471
Hyphomicrobium	0.004366	0.022300
Formosa	0.004878	0.022913
Fusobacterium	0.005040	0.022913
Arenibacter	0.005040	0.022913
Maritimimonas	0.005127	0.022913
AJ224942_g	0.005792	0.025098
*Mycoplasma*_g18	0.006763	0.028224
Lentilitoribacter	0.006908	0.028224
Uc_Cellvibrionales	0.007386	0.029337
Pseudofulvibacter	0.007908	0.030563
Pseudoalteromonas	0.008880	0.033417

Uc_FQ032818_f	0.011672	0.042797
Bizionia	0.013750	0.048709
Lacinutrix	0.013966	0.048709

Table S10. Biomass proportions of algal composition at each site.

Region	Biomass percentage (%)			References
	Chlorophyta	Phaenophyta	Rhdophyta	
Goseong	1.95	82.19	15.87	S1
Pohang	11.10	64.30	24.60	S1,2
Tongyeong	7.89	63.23	28.88	S3
Yeosu	0.10	93.01	6.89	S3,4

Table S11. Significantly different gut microbiota genera between mild and severe barren regions identified by the random forest model.

Genus	*p-value*	*q-value*
Mycobacterium	0.000000118	0.000018032
Colwellia	0.000001374	0.000081165
Psychromonas	0.000001591	0.000081165
Shewanella	0.000002281	0.000087234
FQ032818_g	0.000003182	0.000097360
DQ396288_g	0.000011680	0.000297833
Alkalimarinus	0.000056402	0.000909805
DQ395870_g	0.000079368	0.001103935
EU804292_g	0.000191075	0.002436205
FR685471_g	0.000314356	0.003606639
JN662053_g	0.000999695	0.008201909
Rubritalea	0.009654313	0.035169284

Table S12. Twenty-eight KO gene families overlapping between MaAsLin2 results and features selected by random forest.

Genefamily	KEGG category			Enriched group (Mild vs. Severe)
	3rd	2nd	1st	
K01057: PLGS, 6-phosphogluconolactonase [EC: 3.1.1.31]	Pentose phosphate pathway	Carbohydrate metabolism	Metabolism	Mild
K00175: korB, oorB, oforB, 2-oxoglutarate/2-oxoacid ferredoxin oxidoreductase subunit beta [EC: 1.2.7.3 1.2.7.11]	Glycolysis / Gluconeogenesis	Carbohydrate metabolism	Metabolism	Mild
	Citrate cycle (TCA cycle)	Carbohydrate metabolism	Metabolism	
	Pyruvate metabolism	Carbohydrate metabolism	Metabolism	
	Butanoate metabolism	Carbohydrate metabolism	Metabolism	
	Other carbon fixation pathways	Energy metabolism	Metabolism	
K00334: nuoE, NADH-quinone oxidoreductase subunit E [EC: 7.1.1.2]	Oxidative phosphorylation	Energy metabolism	Metabolism	Mild
K00336: nuoG, NADH-quinone oxidoreductase subunit G [EC: 7.1.1.2]	Oxidative phosphorylation	Energy metabolism	Metabolism	Mild
K01646: citD, citrate lyase subunit gamma (acyl carrier protein)	Two-component system	Signal transduction	Environmental Information Processing	Mild
K07793: tctA, putative tricarboxylic transport membrane protein	Two-component system	Signal transduction	Environmental Information Processing	Mild
K02000: proV, glycine betaine/proline transport system ATP-binding protein [EC: 7.6.2.9]	ABC transporters	Membrane transport	Environmental Information Processing	Mild
K02002: proX, glycine betaine/proline transport system substrate-binding protein	ABC transporters	Membrane transport	Environmental Information Processing	Mild
K02010: afuC, fbpC, iron(III) transport system ATP-binding protein [EC: 7.2.2.7]	ABC transporters	Membrane transport	Environmental Information Processing	Mild
K06726: rbsD, D-ribose pyranase [EC: 5.4.99.62]	ABC transporters	Membrane transport	Environmental Information Processing	Mild

K09816: znuB, zinc transport system permease protein	ABC transporters	Membrane transport	Environmental Information Processing	Mild
K09817: znuC, zinc transport system ATP-binding protein [EC: 7.2.2.20]	ABC transporters	Membrane transport	Environmental Information Processing	Mild
K15770: cycB, ganO, mdxE, arabinogalactan oligomer/maltooligosaccharide transport system substrate-binding protein	ABC transporters	Membrane transport	Environmental Information Processing	Mild
K03568: tldD, TldD protein	Peptidase and inhibitors	Protein family: metabolism	Brite Hierarchies	Mild
K04087: hflC, modulator of FtsH protease HflC	Peptidase and inhibitors	Protein family: metabolism	Brite Hierarchies	Mild
K02026: ycjP, muliple sugar transport system permease protein	Transporters	Protein family: signaling and cellular processes	Brite Hierarchies	Mild
K02069: STAR2, fetB, UDP-glucose/iron transport system permease protein	Transporters	Protein family: signaling and cellular processes	Brite Hierarchies	Mild
K02499: yabN, tetrapyrrole methylase family protein/MazG family protein	Chromosome and associated proteins	Protein family: genetic information processing	Brite Hierarchies	Mild
K06412: spoVG, stage V sporulation protein G	Cell growth	Unclassified: signaling and cellular processes	Not Included in Pathway or Brite	Mild
K07133: uncharacterized protein	Function unknown	Poorly characterized	Not Included in Pathway or Brite	Mild
K00138: aldB, aldehyde dehydrogenase [EC: 1.2.1.-]	Glycolysis / Gluconeogenesis	Carbohydrate metabolism	Metabolism	Severe
	Pyruvate metabolism	Carbohydrate metabolism	Metabolism	
K14153: thiDE, hydroxymethylpyrimidine kinase/phosphomethylpyrimidine kinase/thiamine-phosphate diphosphorylase [EC: 2.7.1.49 2.7.4.7 2.5.1.3]	Thiamine metabolism	Metabolism of cofactors and vitamins	Metabolism	Severe
K06080: rcsF, RcsF protein	Two-component system	Signal transduction	Environmental Information Processing	Severe
K02481: flgR, two-component system, NtrC family, response regulator	Two-component system	Protein family: signaling and cellular processes	Brite Hierarchies	Severe
K02674: pilY1, type IV pilus assembly protein PilY1	Pilus system	Bacterial motility proteins	Brite Hierarchies	Severe
K08095: CUT1_2_3, cutinase	Carboxylic-ester bonds	Unclassified: metabolism	Not Included in Pathway or Brite	Severe
K07484: transposase	Replication and repair	Unclassified: genetic information processing	Not Included in Pathway or Brite	Severe

K14059: int, integrase	Replication and repair	Unclassified: genetic information processing	Not Included in Pathway or Brite	Severe

Supplementary References

S1. Jung SW, Oh YS, Rho HS, Choi CG. 2020. Subtidal marine algal community and endangered species in Dokdo and Ulleungdo, two oceanic islands in the east sea of Kore. Ocean Sci J. 55: 537-547.

S2. Choi CG, Kwon CJ, Kim MK. 2014. Summer marine algal communities at Dokdo, Korea. J Fish Mar Sci Edu. 26: 1037-1043. (Korean)

S3. Choi CG. 2008. Algal flora in Hallyeo-haesang National Park, Southern Coast of Korea. Korean J Fish Aqua Sci. 41: 371-380. (Korean)

S4. Park MS, Yoo HI, Heo JS, Kim YD, Choi HG. 2011. Seasonal variation in the marine algal flora and community structure along the Tongyeong Coast, Korea. Korean J Fish Aqua Sci. 44: 732739. (Korean)
